# Two virulent bacteriophages targeting carbapenem-resistant *Raoultella planticola*

**DOI:** 10.3389/fmicb.2025.1726803

**Published:** 2026-01-09

**Authors:** Cuong V. Hoang, Jonathan Fan, Lauren Bhasin, Anthony Del Mundo, Vanessa Law, Dominic Nguyen, Suraj Ganiger, Ashley Mansour, Christi L. McElheny, Yohei Doi, Olakunle I. Olawole

**Affiliations:** 1 Department of Microbiology & Plant Pathology, University of California, Riverside, Riverside, CA, United States; 2 Division of Infectious Diseases, University of Pittsburgh School of Medicine, Pittsburgh, PA, United States; 3 Department of Microbiology, Fujita Health University School of Medicine, Toyoake, Aichi, Japan; 4 Department of Infectious Diseases, Fujita Health University School of Medicine, Toyoake, Aichi, Japan

**Keywords:** carbapenem-resistant *Raoultella planticola*, bacteriophage therapy, host range, clinical strains, multidrug-resistant pathogens

## Abstract

Carbapenem-resistant *Raoultella planticola* (CRRP) is an emerging nosocomial pathogen with limited therapeutic options. Here, we describe the comparative characterization of two novel virulent bacteriophages, Macy and Sally, both isolated from the same soil microenvironment. Macy exhibits exceptional lytic potency, with a burst size of 8,375 PFU per infected cell, narrow host specificity, and pronounced biofilm-disrupting activity likely mediated by a putative depolymerase. In contrast, Sally displays a broader host range, infecting both *R. planticola* and *R. ornithinolytica* (including a clinical CRRP isolate), while maintaining moderate lytic activity, notable acid tolerance, and substantial biofilm reduction. SNP analysis revealed that resistant isolates carried mutations in genes linked to surface polysaccharide biosynthesis and LysR-family transcriptional regulation, conferring resistance at a measurable cost to bacterial growth fitness. Genomic and phylogenomic analyses further revealed distinct evolutionary trajectories: Macy is a large (147.8 kb) member of Straboviridae Straboviridae with a mosaic genome related to *Raoultella* phages, whereas Sally is a compact (48.5 kb) Casjensviridae phage that is siphovirus more closely aligned with *Klebsiella* and *Enterobacter* phages. Pangenomic comparisons highlighted Macy’s strain-specific gene expansions versus Sally’s cross-genus homology, emphasizing divergent adaptation strategies. Together, these findings illustrate the complementary therapeutic potential of Macy and Sally and establish a genomic and phenotypic foundation for developing effective phage cocktails against multidrug-resistant Raoultella infections.

## Introduction

The rapid global rise of carbapenem-resistant Gram-negative bacteria represents a critical threat to public health, significantly restricting therapeutic options for the treatment of severe and life-threatening infections (Jean et al., 2022). These organisms are often associated with high morbidity and mortality rates, particularly in healthcare settings where immunocompromised and critically ill patients are at elevated risk. Among the array of emerging multidrug-resistant pathogens, *Raoultella planticola* has garnered increasing attention. Formerly classified within cluster II of the genus *Klebsiella*, *R. planticola* is a Gram-negative, facultatively anaerobic rod that has historically been considered an environmental or low-virulence organism. However, it is now increasingly implicated in opportunistic and hospital-acquired infections, including urinary tract infections, bacteremia, and pneumonia, particularly in patients with underlying comorbidities or invasive medical devices (Zhu et al., 2024; Zou et al., 2020; Wang et al., 2015; Xu et al., 2015). Of particular concern is the ability of *R. planticola* to acquire and harbor carbapenemase-encoding genes, including *bla*_KPC_, *bla*_NDM_, and *bla*_OXA-48_, which confer resistance to carbapenem antibiotics, often considered last-resort treatments for multidrug-resistant infections (Zou et al., 2022). The emergence and dissemination of carbapenem-resistant *R. planticola* (CRRP) strains not only complicate clinical management but also raise the risk of horizontal gene transfer to other pathogenic species, exacerbating the broader antimicrobial resistance crisis (Qu et al., 2015). This growing threat highlights an urgent need for enhanced surveillance, rapid diagnostic tools, and the development of novel antimicrobial agents and therapeutic strategies (Castanheira et al., 2009).

The primary means of managing *R. planticola* infections is by administering antibiotics; however, recent studies have shown high concerns about the rapid development of antibiotics resistant variants. While tigecycline-resistant *R. planticola* clinical isolates have been reported (Li et al., 2022), the majority of reported cases are carbapenem-resistant strains. Carbapenems are broad-spectrum antibiotics often us ed. as the last line of defense against serious bacterial infections, especially those caused by multidrug-resistant bacteria. However, CRRP strains have been reported in China (Qu et al., 2015; Xu et al., 2015; Liu et al., 2019; Chen et al., 2020; Zou et al., 2020; Li et al., 2022; Zou et al., 2022; Wang et al., 2023; Zhu et al., 2024), Taiwan (Tseng et al., 2014; Wang et al., 2015; Huang et al., 2018), Turkey (Atici et al., 2018; Ozbey et al., 2023), Germany (Schauer et al., 2022), and the United States (Castanheira et al., 2009; Atici et al., 2018; Park et al., 2019; Iovleva et al., 2020; Ballash et al., 2021; Ozbey et al., 2023). The rapid development of resistant variants has left *R. planticola* infections with very limited treatment options, with the consequence of prolonged hospital stays, increased risk of complications and spread of resistant bacteria in healthcare settings, posing a significant risk to other patients (Österblad et al., 2012; Pfeifer et al., 2012; Piedra-Carrasco et al., 2017). Therefore, urgent efforts are needed to develop new management strategies to prevent their further dissemination and mitigate the associated public health risks.

In light of this, bacteriophages offer a highly promising and potentially transformative approach to combating the growing threat of multidrug resistance, particularly among encapsulated pathogenic bacteria such as *Raoultella* species. Their inherent characteristics make them compelling therapeutic candidates. For instance, phages exhibit high specificity, targeting only specific bacterial strains, thereby minimizing disruption to the beneficial microbiota. They are also non-toxic to human cells and possess the ability to self-replicate at the site of infection, enhancing their therapeutic effect (Born et al., 2017; Hasanien et al., 2024; Wernicki et al., 2017). Moreover, their natural abundance in the environment also makes them readily available. These attributes collectively position phages as a potentially powerful tool for controlling and eradicating antibiotic-resistant bacterial infections, offering a much-needed alternative to traditional antibiotic therapies. Although multiple studies have demonstrated the effectiveness of lytic phages in targeting various serotypes of multidrug-resistant *Klebsiella pneumoniae* (Hung et al., 2011; Dufour et al., 2015; Anand et al., 2020; Dhungana et al., 2021; Hesse et al., 2021; Ichikawa et al., 2023; Weber-Dabrowska et al., 2023; Gholizadeh et al., 2024; Zhao et al., 2025), research on phage-based studies against *Raoultella* species remains extremely limited, with only a small number of phages reported and even fewer characterized in depth. Existing studies, including two phages isolated from *R. ornithinolytica* in Iran, provided initial isolation data but did not include mechanistic or *in vivo* evaluations needed to establish therapeutic potential (Zamani et al., 2019). Therefore, studies on phage therapy treatment against CRRP remains largely unexplored, and there is a need for well-characterized phages with demonstrated biofilm-disrupting and lytic activity against clinical isolates.

A major driver of multidrug-resistance and bacterial persistence across diverse environments is the biofilm, a community of surface-attached bacterial cells (Andrés-Lasheras et al., 2024). Their complex three-dimensional structure poses a significant challenge in both clinical and food industry settings, as conventional antibiotics struggle to penetrate biofilms embedded within an extracellular polymeric substances (EPS) matrix, thus making the search for effective biofilm eradication strategies crucial (Donlan, 2000). Recent research has highlighted the potential of phage-encoded enzymes, particularly endolysins and depolymerases, as promising alternatives (Hughes et al., 1998; Islam et al., 2024; Oliveira et al., 2019). Endolysins can directly degrade bacterial cell walls, causing rapid lysis, while depolymerases break down the EPS that protect biofilm communities, thereby enhancing phage penetration and bacterial clearance.

Despite the growing clinical recognition of *R. planticola* as an emerging opportunistic pathogen, including strains exhibiting carbapenem resistance, there remains a critical lack of targeted therapeutic options, and phage-based interventions for this genus are still poorly explored. Existing studies have identified *Raoultella* phages, yet their therapeutic potential has not been systematically evaluated, leaving a substantial gap in alternative treatments for multidrug-resistant infections. Therefore, the aim of this study was to isolate and characterize new lytic bacteriophages active against carbapenem-resistant *R. planticola*, assess their host range and biofilm-disrupting capacity, and evaluate their genomic suitability for therapeutic development. By establishing foundational biological and genomic profiles of these candidate phages, this work provides essential groundwork for the rational design of future therapeutic phage cocktails targeting CRRP.

## Materials and methods

### Bacterial and phage isolation

Soil samples were collected from the University of California, Riverside nematology greenhouse for bacterial and phage isolation. For bacterial isolation, soil samples were agitated in double-strength LB broth at 30 °C and 280 rpm for 2 h. Suspensions were serially diluted and plated on LB agar to obtain individual colonies. Bacterial isolates were initially identified based on colony morphology and confirmed by 16S rRNA gene sequencing. We focused on a *Raoultella* strain, which was further validated through whole-genome sequencing. This strain was subsequently used as the host for phage isolation. The remaining soil suspension was centrifuged at 7,000 rpm, and the supernatant was filter-sterilized using 0.22 μm pore filters to collect soil filtrates. Filtrates were enriched overnight with a cocktail of all isolated bacterial strains in a 250 mL flask at 30 °C and 280 rpm. After centrifugation (7,000 rpm) and filtration, the enriched supernatants were spotted onto overlays of each bacterial isolate and incubated overnight at 30 °C. Plaques showing clear lysis were picked and resuspended in phage SM buffer (50 mM Tris–HCl, 100 mM NaCl, 8 mM MgSO₄, 0.01% gelatin). Phages were further purified by three rounds of double-layer agar overlay on their respective hosts and stored at −80 °C for long-term preservation.

### Antibiotics susceptibility

A two-fold serial dilution of antibiotic was prepared in sterile LB broth, spanning an appropriate concentration range. Each well of a 96-well plate contained 50 μL of antibiotic dilution and 50 μL of LB broth. A standardized bacterial suspension of *R. planticola* RP8, adjusted to a turbidity of 1.6, was added at 50 μL per well, resulting in a final well volume of 150 μL. Control wells included a growth control (bacteria without antibiotic) and a sterility control (LB only). Plates were incubated at 30 °C for 48 h with intermittent shaking in a SpectraMax iD3 multi-mode microplate reader (Molecular Devices, San Jose, CA, United States). Absorbance readings were collected every 30 min, and wells were visually inspected at the end of incubation to assess bacterial growth.

### Host range assay

Multiple strains of *R. planticola* and *R. ornithinolytica* of clinical origin (Table 1) were used to evaluate phage host range. Bacterial lawns were prepared by mixing 100 μL of overnight culture, standardized to an OD₆₀₀ of 0.8, with 5 mL of molten 0.5% top agar, which was then overlaid on LB agar plates. High-titer phage suspensions (15 μL) were spotted onto the bacterial lawns. Plates were incubated at 28 °C, and lysis zones were recorded after overnight incubation.

**Table 1:** Lists of bacterial strains and their sensitivity to Macy and Sally.

Bacterial strain	Isolate ID	Source	Sensitivity to Macy	Sensitivity to Sally
*Raoultella planticola* ^a^	RP8	This study	+	+
*Raoultella planticola type strain* ^b^	ATCC 700831	ATCC	−	+
*Raoultella ornithinolytica type strain* ^b^	ATCC 31898	ATCC	−	+
*Klebsiella pneumoniae type strain* ^b^	ATCC 13883	ATCC	−	−
*Klebsiella oxytoca type strain* ^b^	ATCC 49473	ATCC	−	−
*Klebsiella oxytoca/Raoultella species* ^b^	35	University of Pittsburgh School of Medicine	−	−
*Klebsiella oxytoca/Raoultella species* ^b^	84	University of Pittsburgh School of Medicine	−	−
*Klebsiella oxytoca/Raoultella species* ^b^	148	University of Pittsburgh School of Medicine	−	−
*Klebsiella oxytoca/Raoultella species* ^b^	674	University of Pittsburgh School of Medicine	−	−
*Klebsiella oxytoca/Raoultella species* ^b^	1,039	University of Pittsburgh School of Medicine	−	−
*Klebsiella oxytoca/Raoultella species* ^b^	1,110	University of Pittsburgh School of Medicine	−	−
*Klebsiella oxytoca/Raoultella species* ^b^	1,128	University of Pittsburgh School of Medicine	−	−
*Klebsiella oxytoca/Raoultella species* ^b^	1,208	University of Pittsburgh School of Medicine	−	−
*Klebsiella oxytoca/Raoultella species* ^b^	1,210	University of Pittsburgh School of Medicine	−	−
*Klebsiella oxytoca/Raoultella species* ^b^	1,213	University of Pittsburgh School of Medicine	−	−
*Klebsiella oxytoca/Raoultella species* ^b^	1,227	University of Pittsburgh School of Medicine	−	−
*Klebsiella oxytoca/Raoultella species* ^b^	1,276	University of Pittsburgh School of Medicine	−	−
*Klebsiella oxytoca/Raoultella species* ^b^	1,590	University of Pittsburgh School of Medicine	−	−
*Klebsiella oxytoca/Raoultella species* ^b^	1,688	University of Pittsburgh School of Medicine	−	−
*Klebsiella oxytoca/Raoultella species* ^b^	1711	University of Pittsburgh School of Medicine	−	−
*Klebsiella oxytoca/Raoultella species* ^b^	1897	University of Pittsburgh School of Medicine	−	−
*Klebsiella oxytoca/Raoultella species* ^b^	1901	University of Pittsburgh School of Medicine	−	−
*Klebsiella oxytoca/Raoultella species* ^b^	1902	University of Pittsburgh School of Medicine	−	−
*Klebsiella oxytoca/Raoultella species* ^b^	1942	University of Pittsburgh School of Medicine	−	−
*Klebsiella oxytoca/Raoultella species* ^b^	1953	University of Pittsburgh School of Medicine	−	−
*Klebsiella oxytoca/Raoultella species* ^b^	1983	University of Pittsburgh School of Medicine	−	−
*Klebsiella oxytoca/Raoultella species* ^b^	2067	University of Pittsburgh School of Medicine	−	−
*Klebsiella oxytoca/Raoultella species* ^b^	2090	University of Pittsburgh School of Medicine	−	+
*Klebsiella oxytoca/Raoultella species* ^b^	2,111	University of Pittsburgh School of Medicine	−	−
*Klebsiella oxytoca/Raoultella species* ^b^	2,141	University of Pittsburgh School of Medicine	−	−
*Klebsiella oxytoca/Raoultella species* ^b^	2,160	University of Pittsburgh School of Medicine	−	−
*Klebsiella oxytoca/Raoultella species* ^b^	2,309	University of Pittsburgh School of Medicine	−	−
*Klebsiella oxytoca/Raoultella species* ^b^	2,395	University of Pittsburgh School of Medicine	−	−
*Klebsiella oxytoca/Raoultella species* ^b^	2,407	University of Pittsburgh School of Medicine	−	−
*Klebsiella oxytoca/Raoultella species* ^b^	2,565	University of Pittsburgh School of Medicine	−	−
*Klebsiella oxytoca/Raoultella species* ^b^	2,687	University of Pittsburgh School of Medicine	−	−
*Klebsiella oxytoca/Raoultella species* ^b^	2,711	University of Pittsburgh School of Medicine	−	−
*Klebsiella oxytoca/Raoultella species* ^b^	2,798	University of Pittsburgh School of Medicine	−	−
*Klebsiella oxytoca/Raoultella species* ^b^	2,840	University of Pittsburgh School of Medicine	−	−
*Klebsiella oxytoca/Raoultella species* ^b^	2,938	University of Pittsburgh School of Medicine	−	−
*Klebsiella oxytoca/Raoultella species* ^b^	2,973	University of Pittsburgh School of Medicine	−	−
*Raoultella ornithinolytica* ^b^	YDC775-5	University of Pittsburgh School of Medicine	−	−
*Raoultella ornithinolytica* ^b^	YDC775-9	University of Pittsburgh School of Medicine	−	−
*Raoultella ornithinolytica* ^b^	YDC775-15	University of Pittsburgh School of Medicine	−	−

^a^Environmental isolates; ^b^Clinical isolates.

### Stability assays

For thermal stability assay, the Sally and Macy phage samples (10^6^–10^7^ PFU/mL) were incubated at 30, 40, 50, 60, 70 or 80 °C for 1 h in a thermocycler. Samples were collected at 6, 12, 18, and 24 h post-incubation for the time-course temperature stability test. For pH stability assay, SM buffer was standardized to different pH values (1, 3, 5, 7, 9, 11, or 13) using 1 M HCl or 1 M NaOH. A 10-fold serial dilution of each phage sample was made with the pH-standardized solutions and incubated at 37 °C for 1 h. Heat- and pH-treated samples were serially diluted and relevant dilutions were titered on double-layer agar overlays. Treatments were performed in triplicates and each experiment repeated at least twice.

### Multiplicity of infection

Bacterial and phage titers (CFU/mL and PFU/mL, respectively) were used to prepare phage-bacterial suspensions at MOIs of 100, 10, 1, 0.1, 0.01, 0.001, and 0.0001. Each MOI group was 10-fold serially diluted in LB broth and incubated at 30 °C for 5 h with shaking at 180 rpm. Lysates were subsequently serially diluted and titrated on double-layer agar overlays. All treatments were performed in triplicate, and experiments were repeated at least twice. Values are presented as the average of all collected readings. The MOI yielding the highest final phage titer was designated as optimal and used for all downstream applications.

### One-step growth curve

To determine phage burst size, latent period, and burst time, phage-bacterial suspensions were prepared at the optimal MOI in 10 mL volumes. Suspensions were incubated at 30 °C for 10 min without shaking and then centrifuged at 10,000 rpm for 10 min. Pellets were resuspended in prewarmed 10 mL LB and incubated at 30 °C with shaking at 160 rpm. Samples were collected every 5 min for the first 50 min and every 10 min for an additional 100 min. Collected samples were serially diluted and titrated using double-layer agar overlays. All treatments were performed in triplicate, and experiments were repeated at least twice.

### Phage adsorption rate assay

Phage-bacterial suspensions were prepared at the optimal MOI and incubated at 30 °C with shaking at 120 rpm. Samples (500 μL) were collected every 2 min for 20 min and centrifuged at 10,000 rpm for 2 min. The supernatant containing unadsorbed phages was filter-sterilized using 0.22 μm filters. Filtrates were serially diluted to determine the titer of free phages. All treatments were performed in triplicate, and experiments were repeated at least twice. The percentage of phage adsorption at different time points was calculated as: % Adsorption = (Initial PFU – free phage PFU) × 100/Initial PFU.

### Phage lytic activity against planktonic cells and pre-formed biofilms

Phage-bacterial suspensions were prepared at MOIs of 100, 10, 1, 0.1, 0.01, 0.001, and 0.0001 in a microtiter plate. Samples were incubated at 30 °C for 12 h with intermittent shaking in a SpectraMax iD3 multi-mode microplate reader (Molecular Devices, San Jose, CA, United States). Bacterial growth and media controls were included. Absorbance readings were collected every 30 min. All treatments were performed in triplicate and experiments were repeated at least twice. For biofilm assay, standardized bacterial cells (100 μL) were added to microtiter plate wells and incubated at 30 °C without shaking for 24 h to allow biofilm formation. After 24 h, 100 μL of 10-fold serially diluted phage stock, starting from 10^10^ PFU/mL, was added to pre-formed biofilms and incubated at 30 °C without shaking. After phage treatment, planktonic cells were removed by washing three times with sterile distilled water (SDW). Plates were air-dried, and biofilms were stained with 150 μL of 0.1% (w/v) crystal violet for 20 min at room temperature. Excess stain was removed by three gentle washes with SDW, and biofilms were air-dried, solubilized with 95% ethanol, and absorbance measured at 595 nm. Treatments were performed in triplicate, and experiments were repeated at least twice.

### Assembly and annotation of phage genome sequence

Purified lysates of phages were concentrated by centrifugation, at 7000 g for 17 h at 10 °C. Phage genomic DNA was isolated using a Phage DNA isolation kit (Norgen Biotek; Thorold, Canada), and concentrations were measured using a QuBit DNA Quantification Kit (Life Technologies, Carlsbad, CA). Purified DNA was sequenced on the Illumina MiSeq platform using Illumina v2 500 cycle reagent chemistry, generating paired-end 250 bp reads (Steemers and Gunderson, 2005). Illumina reads were adapter- and quality-trimmed using Trimmomatic v0.39 (Bolger et al., 2014), and the quality of the trimmed reads was assessed using FastQC v0.11.7^1^. High quality reads were pre-processed using FastX Toolkit^2^ and assembled using SPAdes 3.15.5 at k-mer settings of 21,33,55 (Bankevich et al., 2012). Contigs with low coverage were filtered from the assembly to yield a single contig for each phage. The quality and completeness of each genome assembly was evaluated using QUAST v5.1.0 (Gurevich et al., 2013) and BUSCO v5.8.0 (Seppey et al., 2019). The genomic termini of closed genome of Macy and Sally were analyzed using PhageTerm (Garneau et al., 2017). Phage genomes were annotated using Pharokka v1.3.0 (Bouras et al., 2023) followed by manual curation. Coding sequences (CDS) were predicted with PHANOTATE v1.5.1 (McNair et al., 2019) and tRNAs were predicted with tRNAscan-SE v2.0.11 (Chan et al., 2021).

### Comparative genomic, phylogenetic and pangenome analysis of phage genomes

A comparative genome map was constructed using BLAST Ring Image Generator (BRIG), which is a cross-platform application that enables the interactive generation of comparative genomic images via a graphical user interface (Alikhan et al., 2011). The average amino acid identity (AAI) calculator tool (Assis et al., 2017) was used to compare identity between orthologous genes from characterized phages and reference phages. Orthologous genes were extracted, clustered to 30% protein identity and visualized using Clinker and clustermap.js (Gilchrist and Chooi, 2021). Whole genome and amino acid alignments were obtained using Mafft vs7.505 and trees were generated using Fasttree 2.1.11 with 3000 bootstrap replicates; the resulting phylogenies were visualized and annotated using FigTree vs 1.4.4 (Rambaut, 2008). For pangenome analysis, all selected phage genomes were annotated using Pharokka v1.3.0 (Bouras et al., 2023) followed by manual curation. Annotated files from Pharokka were used for a pan-genome analysis using Roary, which compiles orthogroups based on the presence or absence of orthogroup members (Page et al., 2015). The web-based tool, Venny vs 2.1^3^ and UpSet (Lex et al., 2014) were used to quantitatively analyze intersecting gene sets between and within phage genera.

### Transmission electron microscopy and halo phage plaque morphology

Electron microscopy of ultra-centrifuge-purified phage (~1 × 10^10^ PFU/ml) was performed by using phage dilutions made with a 5-fold dilution SM buffer. Diluted phage solutions were pipetted onto thin 400-mesh carbon-coated Formvar grids, stained with 2% (wt/vol) uranyl acetate, and air dried; excess stain solution was gently washed off with sterile distilled water. Specimen-containing grids were observed on a Talos L120C Transmission Electron Microscope operating at an acceleration voltage of 100 kV. To calculate mean values and standard deviations for dimensions of capsid and tail, three virions were measured and averaged, using Image J (Schneider et al., 2012). In addition to the TEM pictures, high dilutions that yielded 1–10 halo plaques were titered. Halo sizes were monitored at 24, 48, 72 and 98 h post-incubation. Pictures of plates were taken, and halo sizes were estimated using ImageJ. Image sizes were compared using students’ *t*-test. Each treatment was performed in triplicates.

### Phage resistance and anti-phage defense mechanisms

Spontaneous phage-resistant mutants of *R. planticola* strain RP8 were generated by repeated exposure to high phage titers of Macy and Sally until resistant colonies emerged. Resistant isolates were purified by streaking and stored at −80 °C for downstream analyses. To assess potential fitness costs, growth dynamics of resistant and wild-type strains were monitored by measuring optical density (OD₆₀₀) every 30 min for 16 h using a SpectraMax iD3 microplate reader (Molecular Devices, USA) at 28 °C. Phage infections were conducted at a multiplicity of infection (MOI) of 0 and 100, where an MOI of 0 corresponds to a no-phage control. Genomic DNA from resistant and wild-type isolates was extracted using the Qiagen DNeasy Blood & Tissue Kit following the manufacturer’s instructions. DNA libraries were prepared and sequenced on the Illumina MiSeq platform to obtain paired-end reads. Raw reads were trimmed and quality-filtered, followed by *de novo* assembly using SPAdes v3.15. SNPs were identified using Snippy v4.6.0^4^, aligning resistant strain reads to the wild-type genome. Variants unique to resistant strains were analyzed, and mutations in genes encoding surface-exposed or membrane-associated proteins were considered candidate phage receptors.

### Statistical analyses

Quantitative data obtained from phage and bacterial plate titrations, microtiter plate absorbance readings, and ImageJ-based measurements were analyzed using GraphPad Prism software (version 9.3.1; GraphPad Software, San Diego, CA, United States). Bacterial growth data, measured as optical density (OD), were used to calculate the area under the growth curve (AUGC). For comparisons between two groups, unpaired two-tailed Student’s *t*-tests were performed. When unequal variances were detected, Welch’s correction was applied. For comparisons involving more than two groups, one-way analysis of variance (ANOVA) was conducted, followed by Tukey’s *post hoc* multiple comparisons test. A *p*-value of <0.05 was considered statistically significant.

## Results

### Isolation, morphology and host range

**Figure 1:**
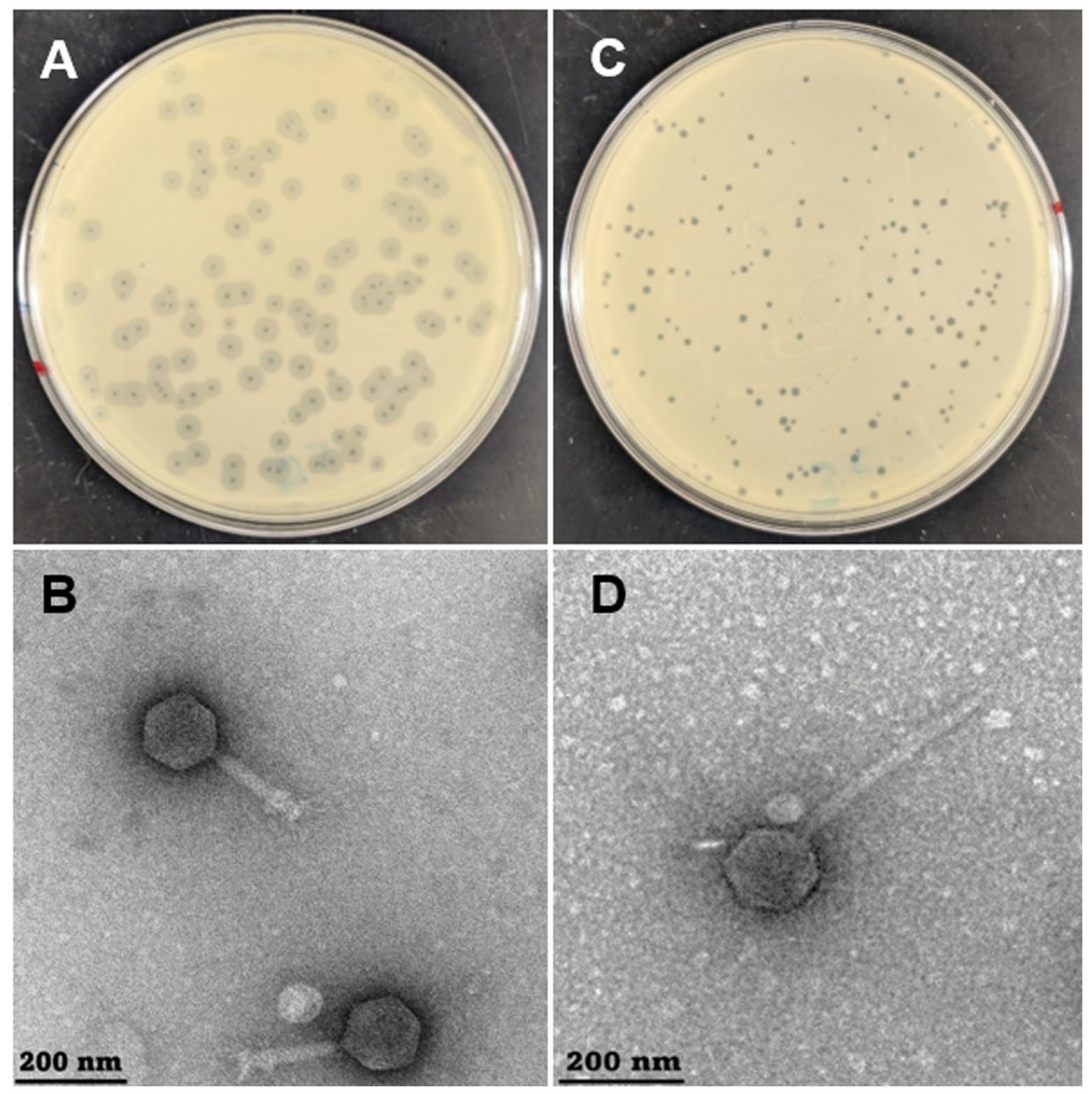
Morphological characteristics of phage Macy and Sally. **(A,C)** Plaques of Macy **(A)** and Sally **(C)** on the host *R. planticola* strain RP8. **(B,D)** Transmission electron microscope images revealing that Macy **(B)** belongs to the Straboviridae Myoviridae family and Sally **(D)** belongs to CasjensviridaeSiphoviridae.

Four distinct bacteriophages were isolated from a soil sample collected near the Nematology greenhouse at the University of California, Riverside. Restriction digest analyses using EcoRV, EcoRI, BamHI, SmaI, and XhoI confirmed that the phages represented unique isolates (Data not shown). Naming was done in accordance with Texas A&M Center for Phage Technology policy, where novel phages are given names for mnemonic purposes. Two of these, designated Sally and Macy, were selected for detailed characterization because they exhibited distinct plaque morphologies and infection profiles compared to the other isolated phages. To evaluate phage activity, all experiments were conducted using the environmental R. planticola strain RP8 as the host. When propagated on overlay lawns of an environmental R. planticola strain RP8, Sally produced clear, circular lytic plaques (Figure 1C), while Macy generated clear plaques surrounded by expanding translucent halos (Figure 1A). The latter suggested the production of extracellular polysaccharide-degrading enzymes, potentially indicative of depolymerase activity and enhanced lytic efficacy. Transmission electron microscopy (TEM) revealed that both phages possess icosahedral capsids and long tails characteristic of the class Caudoviricetes. Macy displayed a compact icosahedral head (55.7 nm) and a shorter, contractile tail (78.6 nm), features consistent with Straboviridae (Myo-like) phages (Figure 1B, Supplementary Table S1). In contrast, Sally exhibited a markedly larger icosahedral head (99.4 nm) and a long, non-contractile tail (265.8 nm), aligning with the morphology of Casjensviridae (Sipho-like) phages (Figure 1D, Supplementary Table S1). Despite their shared overall architecture, these measurements highlight the pronounced structural divergence between the two phages. Host range analyses demonstrated genus-level specificity for both phages. Neither Sally nor Macy exhibited lytic activity against Klebsiella pneumoniae ATCC 13883 or *Klebsiella oxytoca* ATCC 49473 (Table 1). However, their virulence profiles within the Raoultella species differed. Macy infected only *R. planticola* RP8, suggesting a species-specific host range. In contrast, Sally displayed broader activity, infecting *R. planticola* RP8, and the clinical strains *R. planticola* ATCC 700831, *R. ornithinolytica* ATCC 31898, and *Klebsiella oxytoca*/*Raoultella* spp. (Table 1). Together, these results demonstrate that Sally and Macy represent distinct phages with contrasting host ranges and morphologies.

### Antibiotic susceptibility of *R. planticola* strain RP8

**Figure 2:**
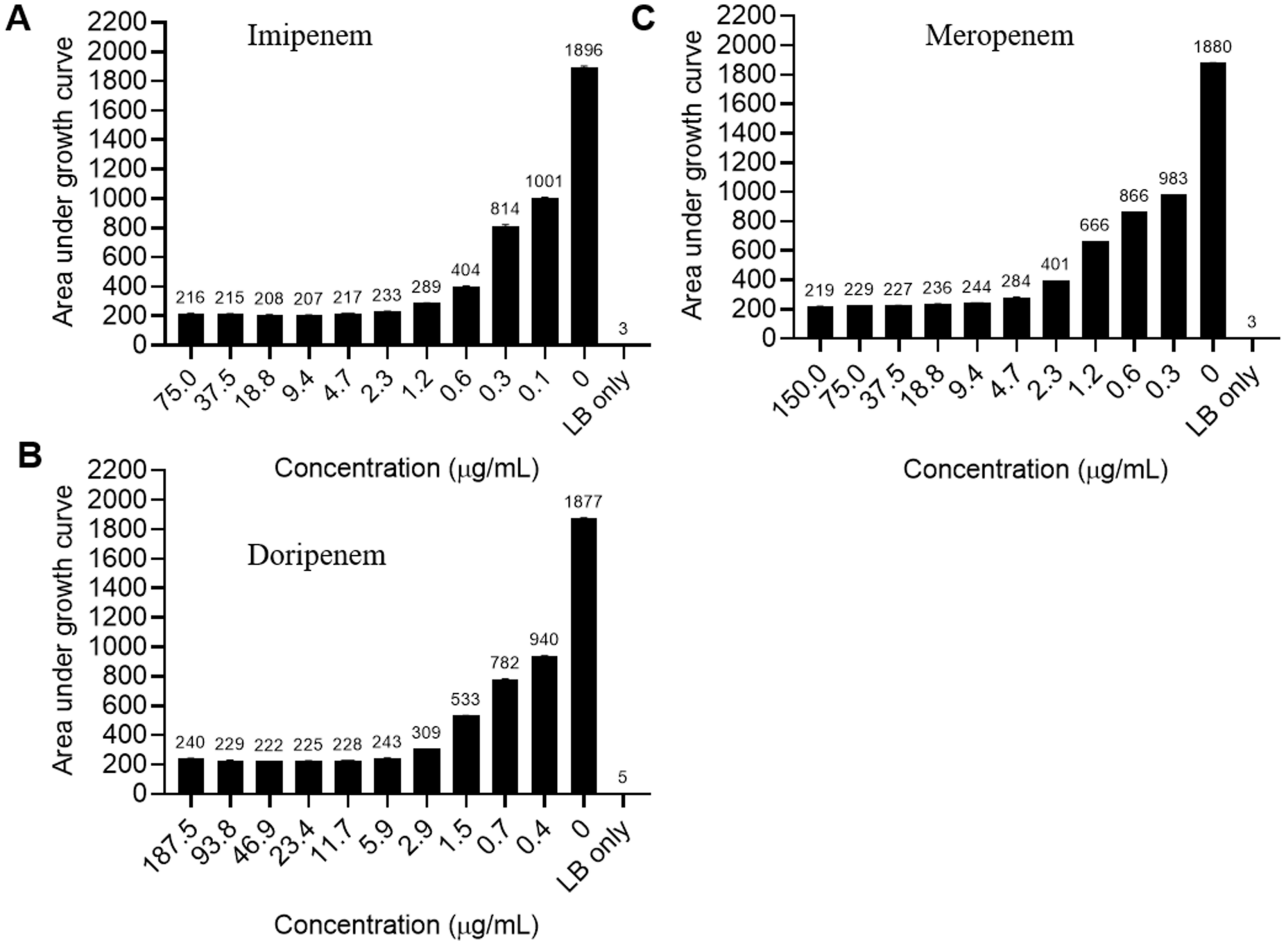
Sensitivity of *Raoultella planticola* strain RP8 to Carbapenem group of antibiotics. Bacterial growth after treatment with different concentrations of Imipenem **(A)**, Doripenem **(B)**, and Meropenem **(C)** for 48 hours at 30°C using a plate reader.

Whole genome sequencing of RP8, despite its environmental origin, revealed the presence of multiple antibiotics and multidrug resistance (MDR) genes (Table 2). Notable resistance genes included six copies of genes encoding multidrug efflux pumps, including multidrug resistance proteins B and MdtO, multiple antibiotic resistance protein MarB, and the MarR transcriptional regulator. Additional resistance determinants included genes for a bleomycin resistance protein, copper resistance protein, and an AraC family multidrug resistance transcriptional activator (Table 2). Therefore, we evaluated the susceptibility of RP8 to selected carbapenems, often considered the last line of defense against resistant infections. Bacterial growth in treated samples exceeded one-tenth of the no-treatment controls at the highest concentrations tested: 75 μg/mL for imipenem, 150 μg/mL for meropenem, and 187.5 μg/mL for doripenem (Figures 2A–C and Supplementary Figure S1). Such robust growth at these elevated antibiotic concentrations highlights the alarming risk of treatment failure and underscores the persistence of MDR infections even under intensive therapeutic pressure.

**Table 2:** Antibiotics and multidrug resistance genes identified in the genome of *Raoultella planticola* strain RP8.

Start	Stop	Strand	Locus Tag	Gene	Product
37495	38184	−	RP8_00185	*azlC*	Putative branched-chain amino acid permease (azaleucine resistance)
364890	365573	−	RP8_01715	*terC*	Tellurite resistance membrane protein TerC
433229	433537	−	RP8_02035	*bsmA*	Biofilm peroxide resistance protein BsmA
594557	594976	+	RP8_02790	*fosA*	FosA family fosfomycin resistance glutathione transferase
598397	599464	+	RP8_02820	*emrA*	Multidrug resistance efflux pump EmrA
960731	961825	−	RP8_04535		Multidrug resistance efflux pump
1005520	1005771	+	RP8_04730	*bhsA*	Multiple stress resistance protein BhsA
1005828	1006166	+	RP8_04735	*araC*	AraC family multidrug resistance transcriptional activator
1180930	1184076	−	RP8_05570	*acrB*	Acriflavine resistance protein B
1184099	1185292	−	RP8_05575	*acrA*	Acriflavine resistance protein A
1209688	1210401	−	RP8_05680	*sapB*	Magnesium uptake protein YhiD/SapB, involved in acid resistance
1281125	1282267	+	RP8_06015		Multidrug resistance efflux pump
2125734	2126105	−	RP8_10105		Glyoxalase/bleomycin resistance protein/dioxygenase
2379356	2380441	−	RP8_11255	*emrA*	Multidrug resistance efflux pump EmrA
2400240	2401388	+	RP8_11340		Fusaric acid resistance family protein
2420496	2421500	+	RP8_11435	*tehA*	Dicarboxylate transporter/tellurite-resistance protein TehA
2421497	2422093	+	RP8_11440	*tehB*	Tellurite resistance methyltransferase TehB
2485191	2485721	+	RP8_11860		Bleomycin resistance protein
2689887	2691173	+	RP8_12820		DHA2 family methylenomycin A resistance protein-like MFS transporter
2767871	2768305	+	RP8_13195	*marR*	Multiple antibiotic resistance transcriptional regulator MarR
2768734	2768940	+	RP8_13205	*marB*	Multiple antibiotic resistance protein MarB
2853798	2854976	−	RP8_13610	*emrA*	Multidrug resistance efflux pump EmrA
2921020	2921916	+	RP8_13940	*emrA*	Multidrug resistance efflux pump EmrA
3292797	3293141	+	RP8_15630	*tehB*	Tellurite resistance protein TehB, SAM-dependent methylase, cupin superfamily
3420152	3420376	+	RP8_16290	*yodD*	YodD family peroxide/acid resistance protein
3625310	3626758	−	RP8_17190	*mdtQ*	Multidrug resistance outer membrane protein MdtQ
4116084	4116830	+	RP8_19435	*azlC*	Putative branched-chain amino acid permease (azaleucine resistance)
4292130	4,292843	+	RP8_20280	*terC*	Tellurite resistance membrane protein TerC
4369809	4371752	+	RP8_20680	*mdtO*	Multidrug resistance protein MdtO
4379023	4379412	−	RP8_20705		Glyoxalase/bleomycin resistance protein/dioxygenase superfamily protein
4410885	4411598	+	RP8_20890	*arsH*	Arsenical resistance protein ArsH
4439266	4439625	+	RP8_21040		Copper resistance protein
4532079	4533611	+	RP8_21505		Multidrug resistance protein B
4638397	4639365	+	RP8_22005	*terC*	Tellurite resistance membrane protein TerC
5106620	5107756	−	RP8_24305	*emrA*	Multidrug resistance efflux pump EmrA

### Optimal multiplicity of infection

Determining the optimal multiplicity of infection (MOI) is essential for maximizing the efficacy of a phage in bacterial control applications. In this study, we evaluated the optimal MOI of Macy and Sally by infecting host bacterial cultures at varying MOIs ranging from 0.0001 to 100. We also assessed bacterial growth inhibition and phage replication efficiency by measuring optical density (OD_600_) and phage titer over time. Our results show that the optimal MOI for phage Macy is 0.001 (Table 3), while the optimal MOI for phage Sally is 1 (Table 4), as these conditions produced the highest burst sizes and most effective bacterial clearance without triggering excessive premature lysis that could limit phage propagation.

**Table 3:** Determination of optimal multiplicity of infection (MOI) of phage Macy.

MOI	Initial concentration		Final concentration
	Bacterial host (CFU/mL)	Phage (PFU/mL)	Phage (PFU/mL)
100	4.90 × 10^8^	5.0 × 10^10^	4.2 × 10^9^
10	4.90 × 10^8^	5.0 × 10^9^	1.5 × 10^8^
1	4.90 × 10^8^	5.0 × 10^8^	2.7 × 10^9^
0.1	4.90 × 10^8^	5.0 × 10^7^	1.1 × 10^10^
0.01	4.90 × 10^8^	5.0 × 10^6^	1.3 × 10^10^
0.001	4.90 × 10^8^	5.0 × 10^5^	2.6 × 10^10^
0.0001	4.90 × 10^8^	5.0 × 10^4^	1.2 × 10^10^

**Table 4:** Determination of optimal multiplicity of infection (MOI) of phage Sally.

MOI	Initial Concentration		Final concentration
	Bacterial host (CFU/mL)	Phage (PFU/mL)	Phage (PFU/mL)
100	4.90 × 10^8^	7.08 × 10^9^	4.18 × 10^8^
10	4.90 × 10^8^	7.08 × 10^8^	2.80 × 10^8^
1	4.90 × 10^8^	7.08 × 10^7^	1.50 × 10^9^
0.1	4.90 × 10^8^	7.08 × 10^6^	5.68 × 10^8^
0.01	4.90 × 10^8^	7.08 × 10^5^	6.23 × 10^8^
0.001	4.90 × 10^8^	7.08 × 10^4^	2.33 × 10^7^
0.0001	4.90 × 10^8^	7.08 × 10^3^	3.13 × 10^8^

### Lytic activity against planktonic and biofilm cells

**Figure 3:**
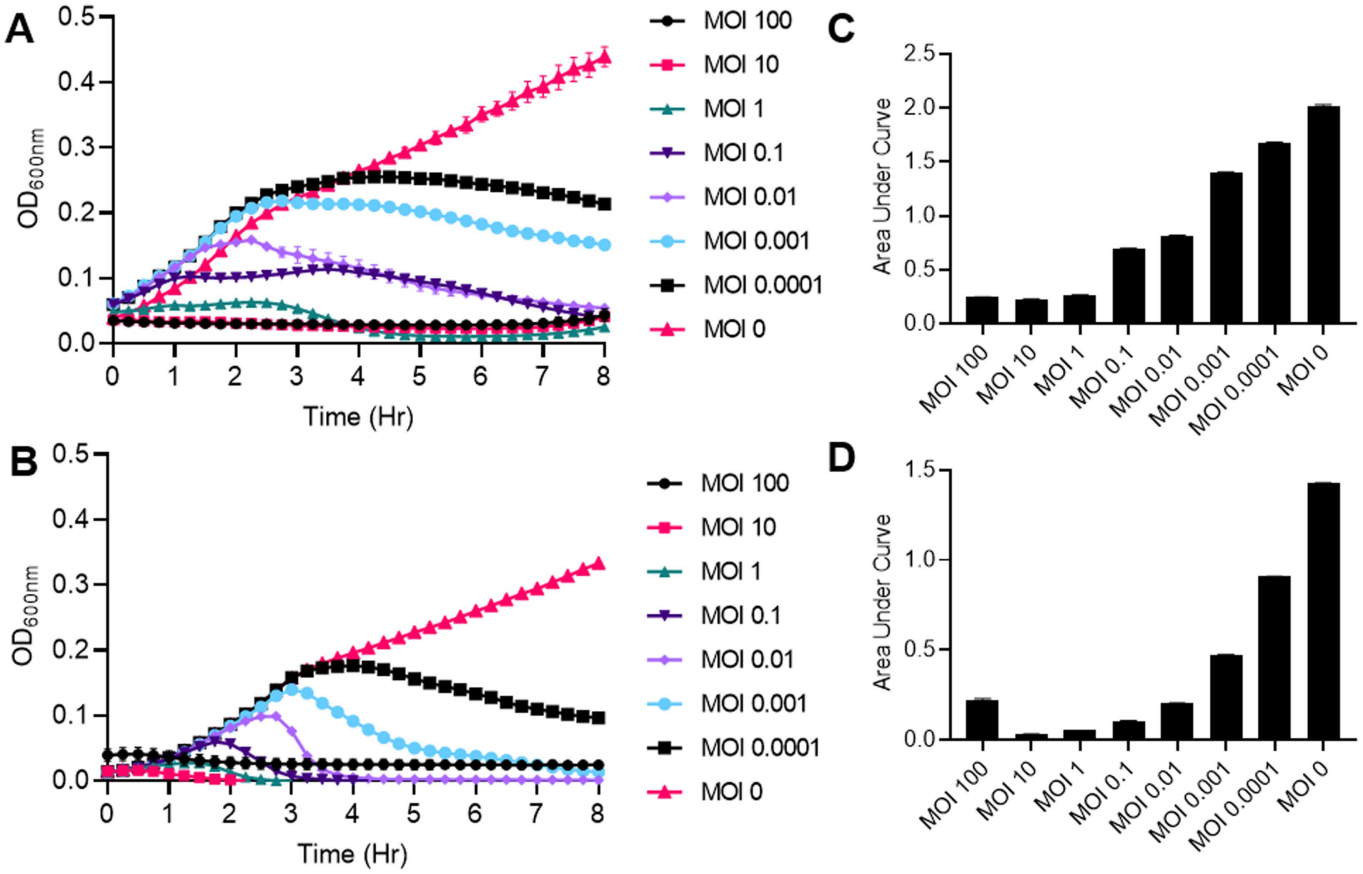
Lytic activity of phage Macy and Sally against planktonic cells. **(A,C)** Bacterial growth curve **(A)** and corresponding area under growth **(C)** of phage Macy. **(B,D)** Bacterial growth curve **(B)** and corresponding area under growth **(D)** of phage SMacy. Bacterial growth was monitored for 8 hours at 30°C using a plate reader.

**Figure 4:**
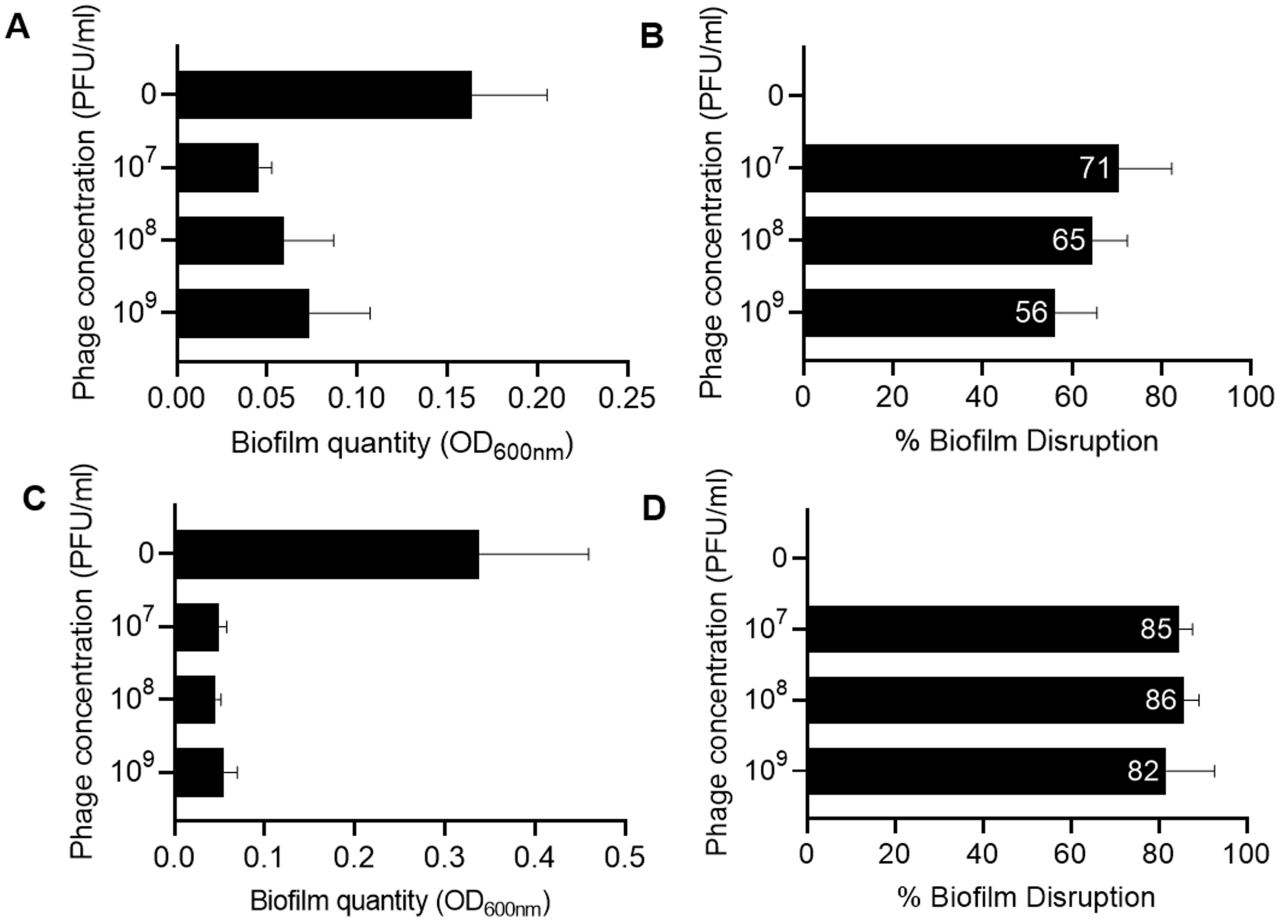
Inhibition and disruption of biofilm formation. **(A,C)** Quantification of biofilm formation by *Raoultella planticola* in the presence or absence of phage Macy **(A)** and Sally **(C)**. Biofilms were stained with 0.1% crystal violet and quantified by measuring absorbance at 595 nm. **(B,D)** Percentage of biofilm disruption by phage Macy **(B)** and Sally **(D)** compared to untreated controls.

Macy and Sally showed MOI-dependent lytic activity, with higher MOIs (1–100) driving faster bacterial reduction but limiting phage amplification due to rapid host depletion (Figures 3A–D). For Macy, an MOI of 0.1 provided an optimal balance with 65% lytic activity and a phage titer of 1.1 × 10^10^ PFU/ml, while for Sally, an MOI of 1 yielded 79% lytic activity and a titer of 1.5 × 10^9^ PFU/ml. Percentage lytic activity refers to the proportion of bacterial growth suppressed in a phage-treated culture compared to an untreated control. Together, these results highlight phage-specific differences in infection dynamics and underscore the importance of optimizing MOI conditions to maximize bacterial suppression and phage propagation. Overall, Macy and Sally demonstrated strong lytic capacity, efficiently lysing planktonic *R. planticola* RP8 cells. For Macy, an MOI of 0.1 provided an optimal balance with 65% lytic activity and a phage titer of 1.1 × 10^10^ PFU/ml, while for Sally, an MOI of 1 yielded 79% lytic activity and a titer of 1.5 × 10^9^ PFU/ml. Together, These results highlight phage-specific differences in infection dynamics and emphasize the need to tailor MOI conditions for maximizing both bacterial suppression and sustainable phage propagation. Together, these results demonstrate that Macy and Sally efficiently lyse planktonic *R. planticola* RP8 cells. Furthermore, Macy demonstrated significant disruption activity against pre-formed biofilm of the *R. planticola* strain RP8. When applied at different concentrations (10^7^–10^9^ PFU/ml), the phage effectively reduced biofilm biomass (Figure 4A), as quantified by crystal violet staining. Compared to untreated controls, phage-treated biofilms showed a 56-71% reduction (Figure 4B). Similarly, Sally exhibited strong biofilm disruption activity against pre-formed RP8 biofilms (Figures 4A–C) and reduced biofilm production by 82–85% (Figure 4D). Together, these findings highlight the potential of phages Macy and Sally as effective biocontrol agents for managing biofilm-associated infections and reducing surface contamination in both clinical and environmental settings.

### Burst size, latent period, and adsorption rate

**Figure 5:**
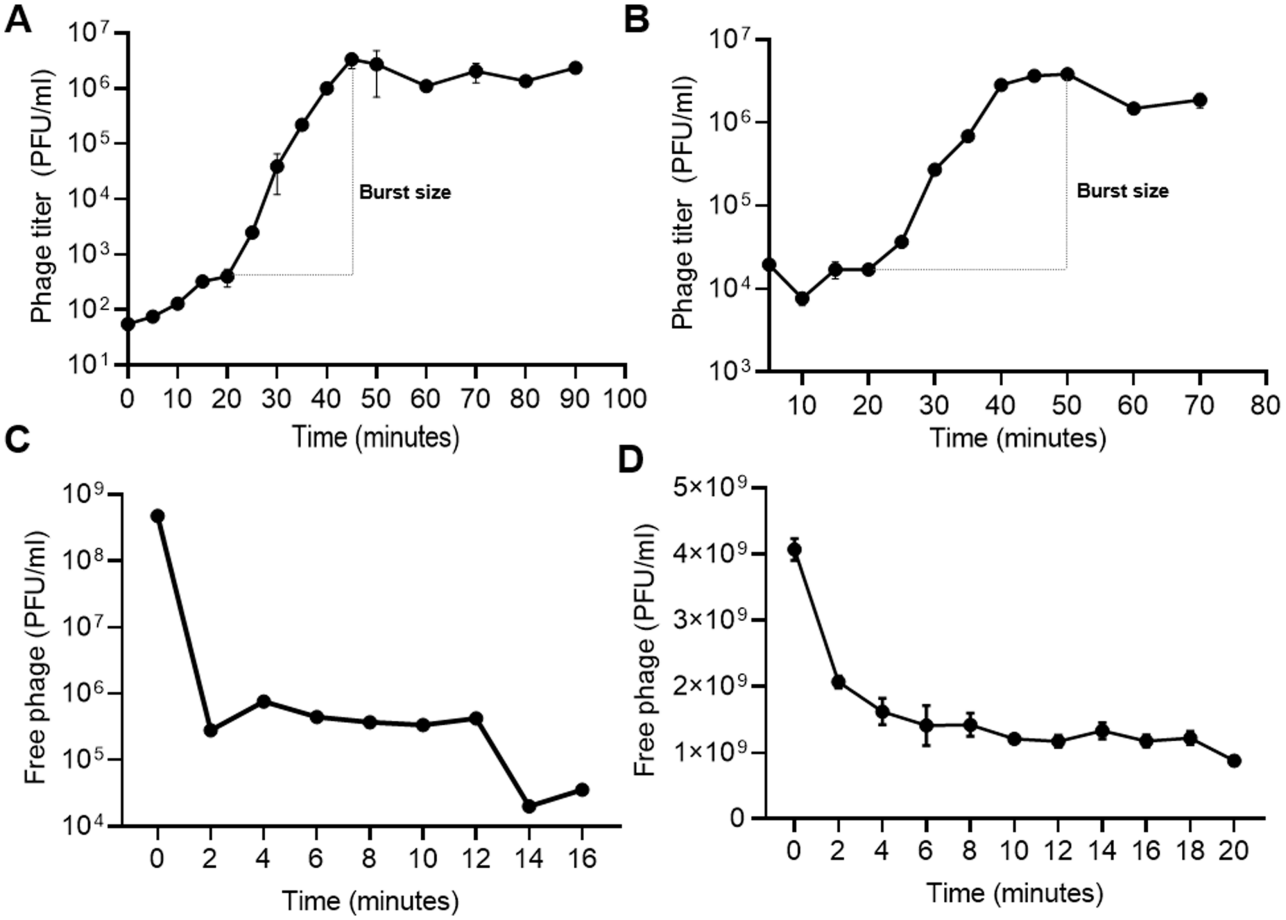
Determination of infection dynamics of phage Macy and Sally. **(A,B)** One step growth curve showing the latent period, burst time and burst size of Macy **(A)** and Sally **(B)**. **(C,D)** Adsorption curve of the phages Macy **(C)** and Sally **(D)**.

To assess the replication dynamics and lytic potential of phages Sally and Macy, one-step growth assays were conducted. Macy exhibited a latent period of 20 min and a burst time of 25 min, producing an exceptionally high burst size with an average release of 8,375 virions per infected cell (Figure 5A). For Sally, the latent period was determined to be 20 min, with a burst time of 30 min and a burst size of 226 virions per infected RP8 cell, relatively lower than that observed for Macy (Figure 5B). Parallel adsorption assays revealed striking differences in binding efficiency, as nearly 50% of Sally particles adsorbed to host cells within 2 min of exposure (Figure 5C), compared to ~99% of Macy virions under the same conditions (Figure 5D). These results indicate moderate receptor-binding efficiency for Sally but exceptionally strong binding for Macy, whose rapid adsorption likely contributes to its large burst size and efficient infection dynamics. Although Macy exhibits a narrower host range and higher strain specificity, its rapid adsorption enables it to initiate infection effectively even at very low MOI.

### Thermal and pH stability

**Figure 6:**
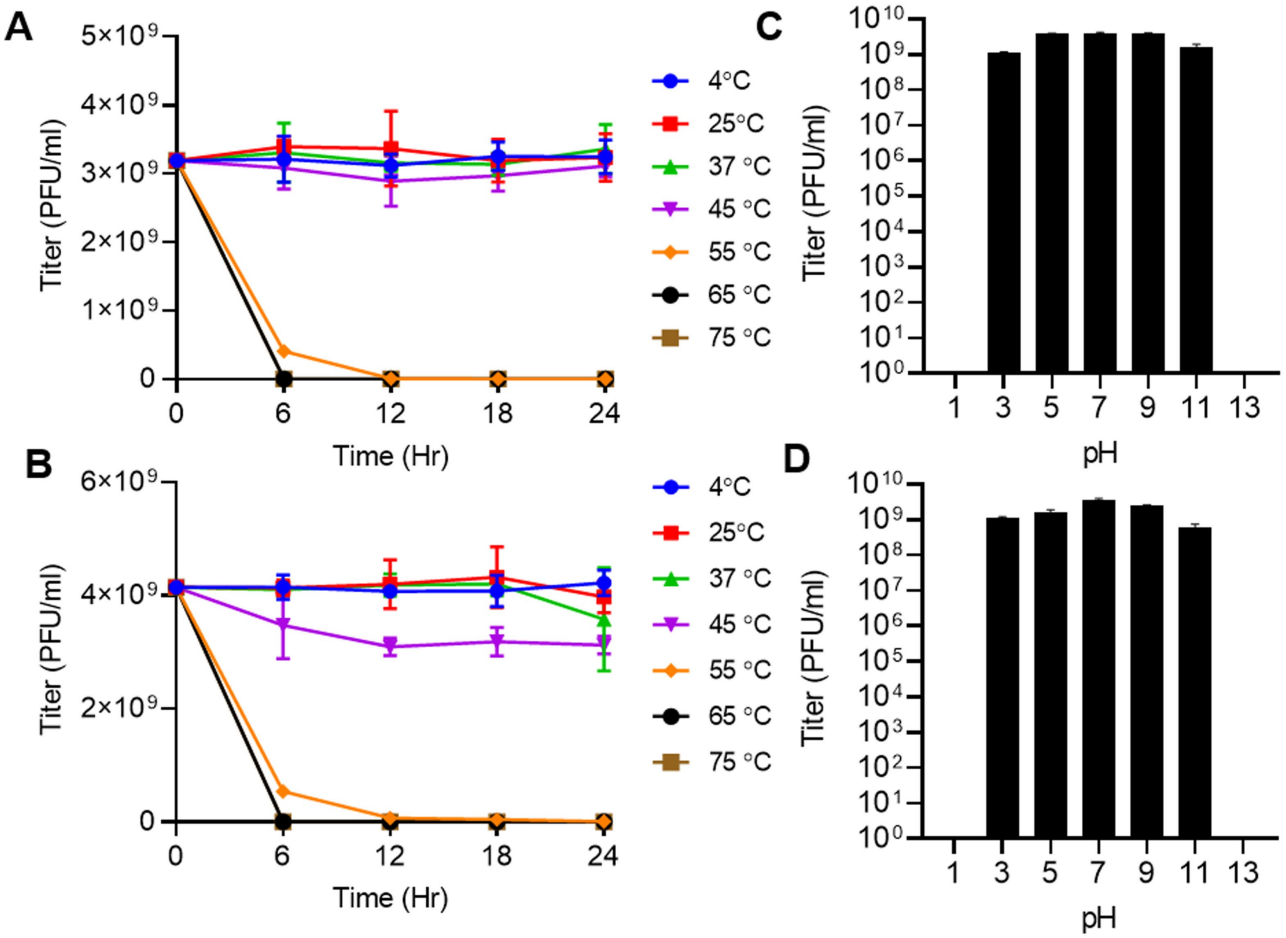
Effect of temperature and pH on the stability of the *Raoultella planticola* phage Macy and Sally. **(A,B)** Influence of temperature on viability of phage Macy **(A)** and Sally **(B)**. Phage suspensions were incubated for 24 hours at different temperature. **(C,D)** Influence of pH on viability of phage Macy **(C)** and Sally **(D)**. Phage suspensions were incubated for one hour at different pH regimes. Each experiment was conducted at least twice.

The stability of Macy and Sally was evaluated under various temperatures and pH conditions to assess its their suitability for downstream applications. Thermal stability tests revealed that both Macy and Sally remained stable over a wide range of temperatures from 4°C to 45°C during a 24-hour incubation period (Figures 6A,B). At temperature higher than 55°C, a significant reduction in infectivity was detected in both phages. pH stability assays showed that Macy and Sally remained infective across a broad range of pH values (3–11), although both lost viability under highly acidic (<3) or strongly alkaline (>11) conditions (Figures 6C,D). This resilience highlights their potential utility in biocontrol applications under variable environmental conditions. This is particularly important for Sally, which infects clinical *Raoultella* strains, suggesting possible therapeutic relevance for gastric or other low-pH infections, where conventional antibiotics are less effective.

### Genomic analysis

**Figure 7:**
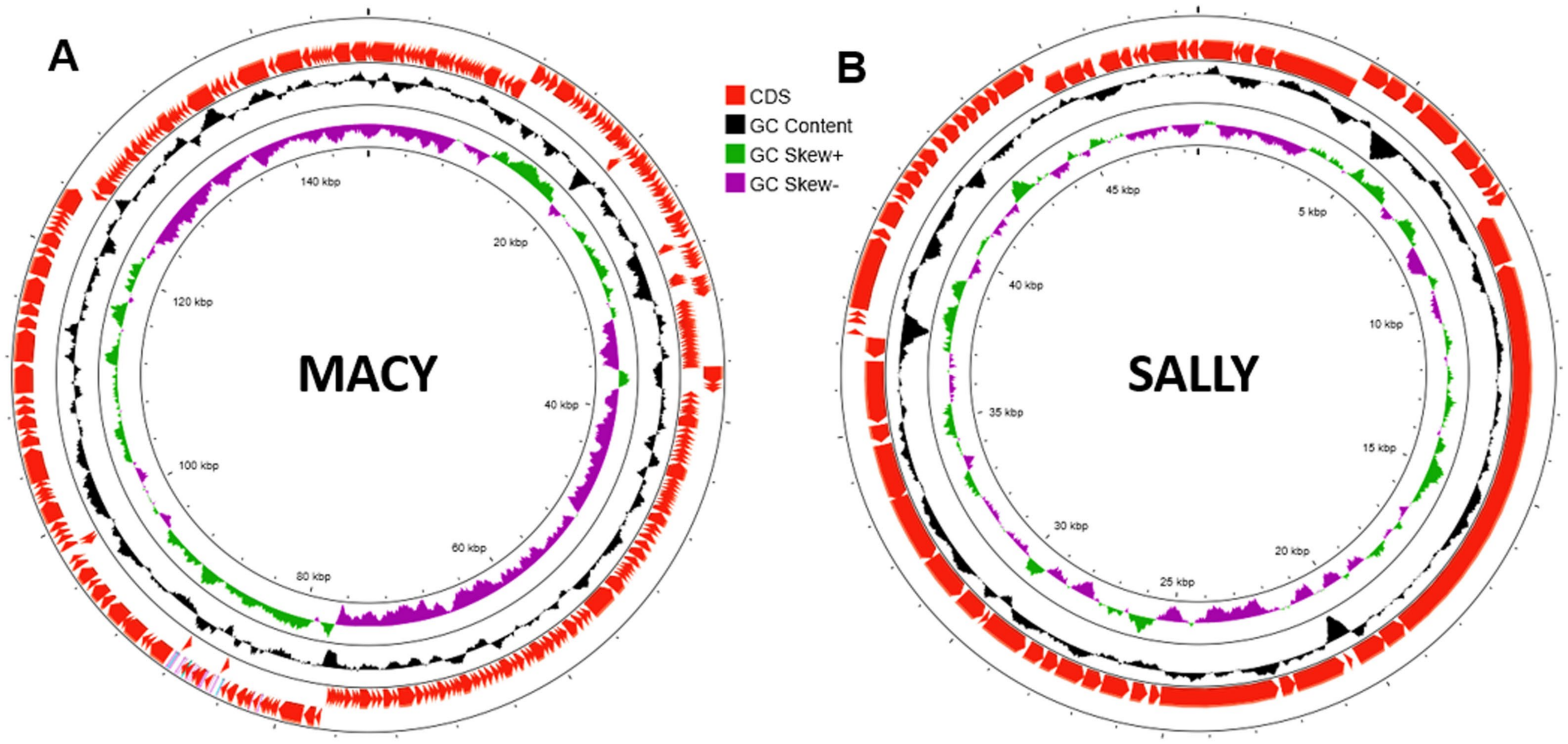
Genomic features of phages Macy and Sally. Circular genome map of phage Macy **(A)** and Sally **(B)**. The genome of Macy is 147.8 kb in length with a GC content of 45% and the genome of Sally is 48.5 kb in length with a GC content of 56%. Open reading frames (ORFs) are color-coded based on predicted functional categories: DNA metabolism, structural proteins (head, tail, connector), lysis proteins, and hypothetical proteins.

**Figure 8:**
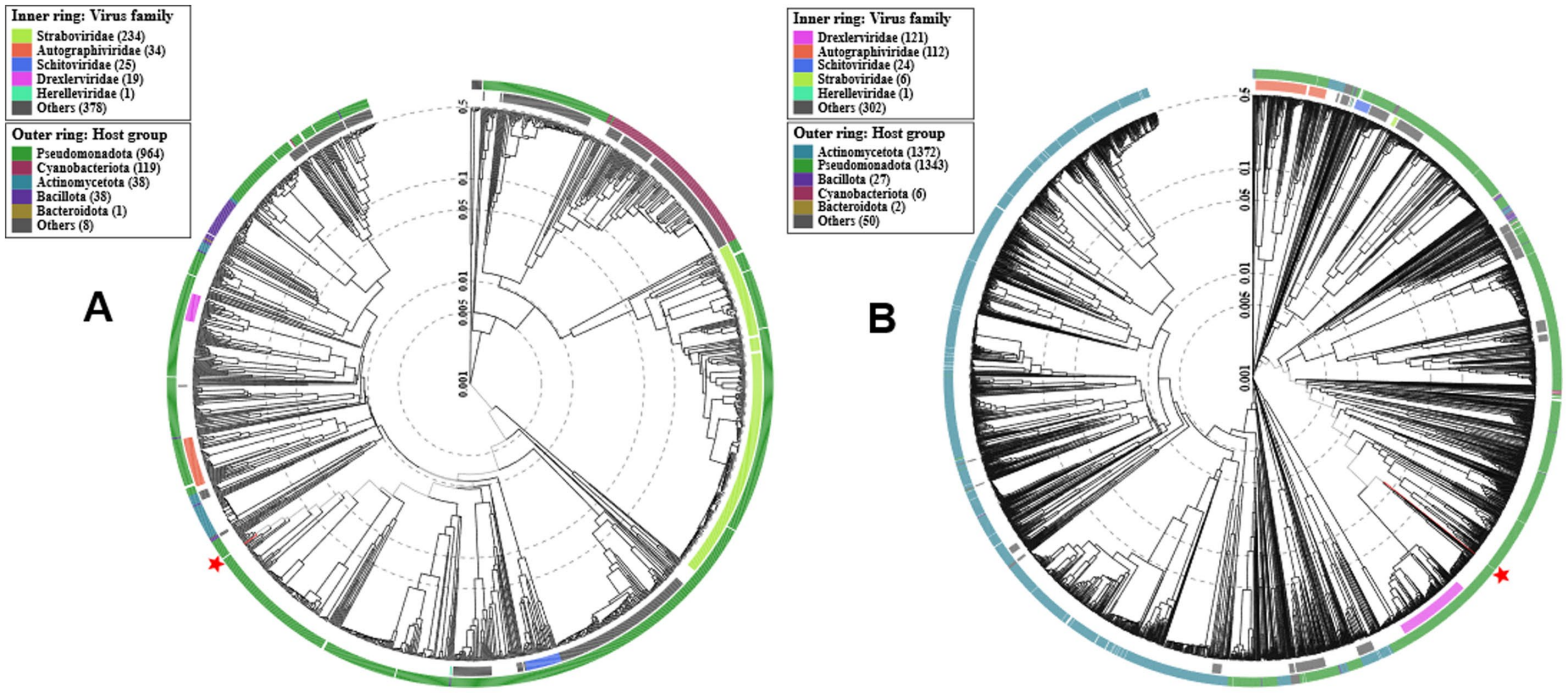
Phylogenetic analysis of phages Macy and Sally. Phylogenetic tree generated using ViPTree based on whole-genome proteomic alignment of phage Macy **(A)** and Sally **(B)** with related members. Asterisks (*) indicate the positions of phages Macy **(A)** and Sally **(B)** within the trees.

**Figure 9:**
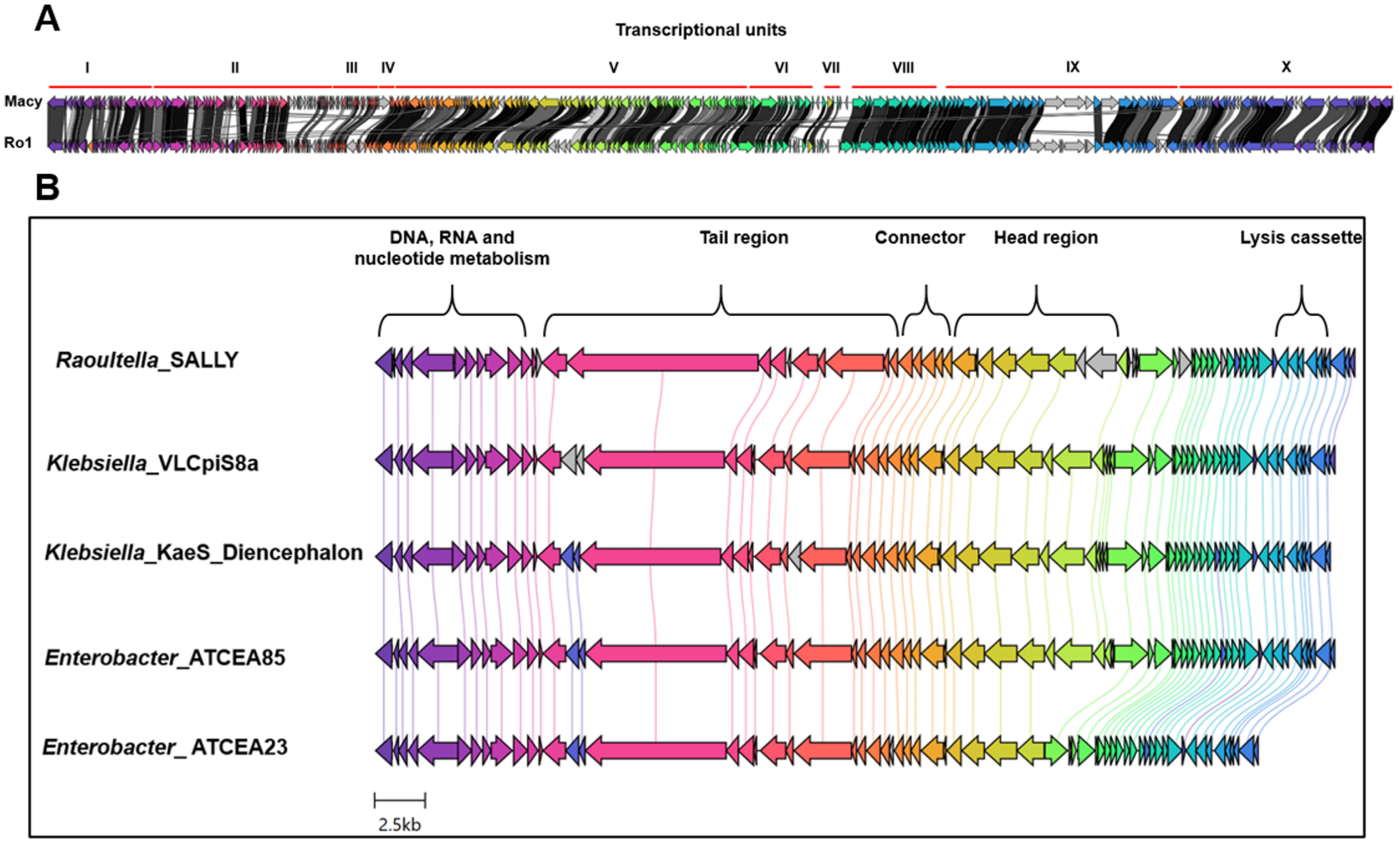
Genome comparison between the *Raoultella planticola* phages and closely related *Klebsiella* and *Enterobacter* phages. **(A)** Comparative topology of the genomes of phages Macy and the closest relative Ro1. Percent amino acid identity represented by greyscale links, and each similarity group is assigned a unique color. **(B)** Synteny illustrating the degree of genomic conservation and gene order similarity of Sally and closely related phages. Open reading frames (ORFs) are color-coded based on predicted functional categories: DNA metabolism, structural proteins (head, tail, connector), lysis proteins, and hypothetical proteins.

Comparative genomics revealed that phages Macy and Sally share only 55% nucleotide similarity (alignment data not shown), with scattered regions of homology, indicating distinct evolutionary origins. Phage Macy has a 147.8 kb genome (291 genes, 45% GC) with 77 bp direct terminal repeats characteristic of T4-like phages (Figure 7A and Supplementary Table S2). ICTV analysis classified Macy within Mydovirus (subfamily Vequintavirinae, family Myoviridae), most similar to *Raoultella* phage Ro1 (Figures 8A, 9A). Despite the relatedness to phage Ro1, Macy diverges substantially at the whole-genome level. A unique feature of Macy is an EPS depolymerase gene (locus tag MACY_240), absent in Ro1, consistent with its halo phenotype and potential role in biofilm disruption (Figure 9A and Supplementary Table S2). Macy and Ro1 share 204 homologous genes with 86.1% average amino acid identity, but exhibit a mosaic genome with at least 10 transcriptional units and 38 terminators, reflecting frequent recombination (Supplementary Figure S2 and Table 5). Phylogenomic analysis placed Macy in the *Klebsiella* II cluster, closely related to clinically relevant *Klebsiella* phages, and protein phylogenies revealed signatures of horizontal gene transfer with *Escherichia*, *Pectobacterium*, and *Serratia* phages (Figure 8A). Pangenome analysis further emphasized divergence between clusters: Macy and Ro1 shared 44% of genes but only 33–37% with *Klebsiella* phages, while Sally shared most core genes with *Klebsiella*/*Enterobacter* phages but retained the highest proportion of strain-specific genes (Supplementary Figure S2 and Table 5).

**Table 5:** Comparative genomic features of Macy and closely related *Raoultella* and *Klebsiella* phages.

Genome features	*Raoultella* phages		*Klebsiella* phages		
	Macy	Ro1	VLCpiM5a	p8734	DP
Accession	This study	NC_048682.1	ON602736.1	PQ133625.1	OP296941.1
Size (bp)	147,807	145,759	141,724	146,651	145,648
GC content (%)	45	44.5	45	45	45
Host GC content (%)	55.5	55.5	57.3	57.3	57.3
% Coding density	93.61	93.23	93.10	93.35	93.29
CDS	291	296	278	289	292
DNA, RNA and nucleotide metabolism	39	43	29	30	31
Head and packaging	9	10	9	9	9
Integration and excision	0	0	0	0	0
Lysis	6	7	6	6	6
Moron, auxiliary metabolic gene and host takeover	8	7	7	7	7
Other	17	18	16	16	16
Tail	18	20	17	18	16
Transcription regulation	1	1	2	2	1
Unknown function	192	189	191	200	205
tRNAs	23	24	24	24	23
CRISPRs	0	0	0	0	0
tmRNAs	0	0	0	0	0
VFDB_Virulence_Factors	0	0	0	0	0
CARD_AMR_Genes	0	0	0	0	0

By contrast, Sally possesses a smaller 48.5 kb genome (66 genes, 56% GC), aligning more closely with its host genome (Figure 7B and Supplementary Table S3). It clustered within the genus *Chivirus* (family Casjensviridae), sharing 80.2% identity with *Klebsiella* phage VLCpiS8a, and was more closely related to *Klebsiella* than *Raoultella* phages (Figures 7B, 8B, 9B). Sally and VLCpiS8a share 21 homologous genes (Supplementary Figure S2). Pangenome analyses revealed a conserved genome organization with *Klebsiella* and *Enterobacter* phages, but Sally encoded the largest number of unique genes within its cluster, highlighting its distinct genetic profile (Figure 9B and Table 6). Together, these results demonstrate that Macy and Sally represent genetically distinct *Raoultella* phages: Macy is a large mosaic T4-like phage with signatures of horizontal gene transfer, while Sally is a compact *Chivirus*-like phage closely allied with *Klebsiella*/*Enterobacter* phages yet carrying unique genes.

**Table 6:** Comparative genomic features of Sally and closely related *Klebsiella* and *Enterobacter* phages.

Genome features	Sally	*Klebsiella* phages		*Enterobacter* phages	
		VLCpiS8a	KaeS_Diencephalon	ATCEA23	ATCEA85
Accession	This study	ON602747.1	OL539440.1	MW419910.1	MN656993.1
Size (bp)	48,479	47,499	47,263	43,692	47,484
GC content (%)	56	56	56	56	56
Host GC content (%)	55.5	57.3	57.3	55.5	55.5
% Coding density	96.65	96.98	97.34	96.76	97.19
CDS	66	66	67	63	66
Connector	4	4	4	5	4
DNA, RNA and nucleotide metabolism	6	4	5	4	4
Head and packaging	5	5	5	4	5
Integration and excision	0	0	0	0	0
Lysis	3	3	3	3	3
Moron, auxiliary metabolic gene and host takeover	0	0	0	0	0
Other	2	2	2	2	2
Tail	10	10	11	10	10
Transcription regulation	1	0	0	0	0
Unknown function	35	38	37	35	38
tRNAs	0	0	0	0	0
CRISPRs	0	0	0	0	0
tmRNAs	0	0	0	0	0
VFDB_Virulence_Factors	0	0	0	0	0
CARD_AMR_Genes	0	0	0	0	0

### Phage resistance and anti-phage defense mechanisms

**Figure 10:**
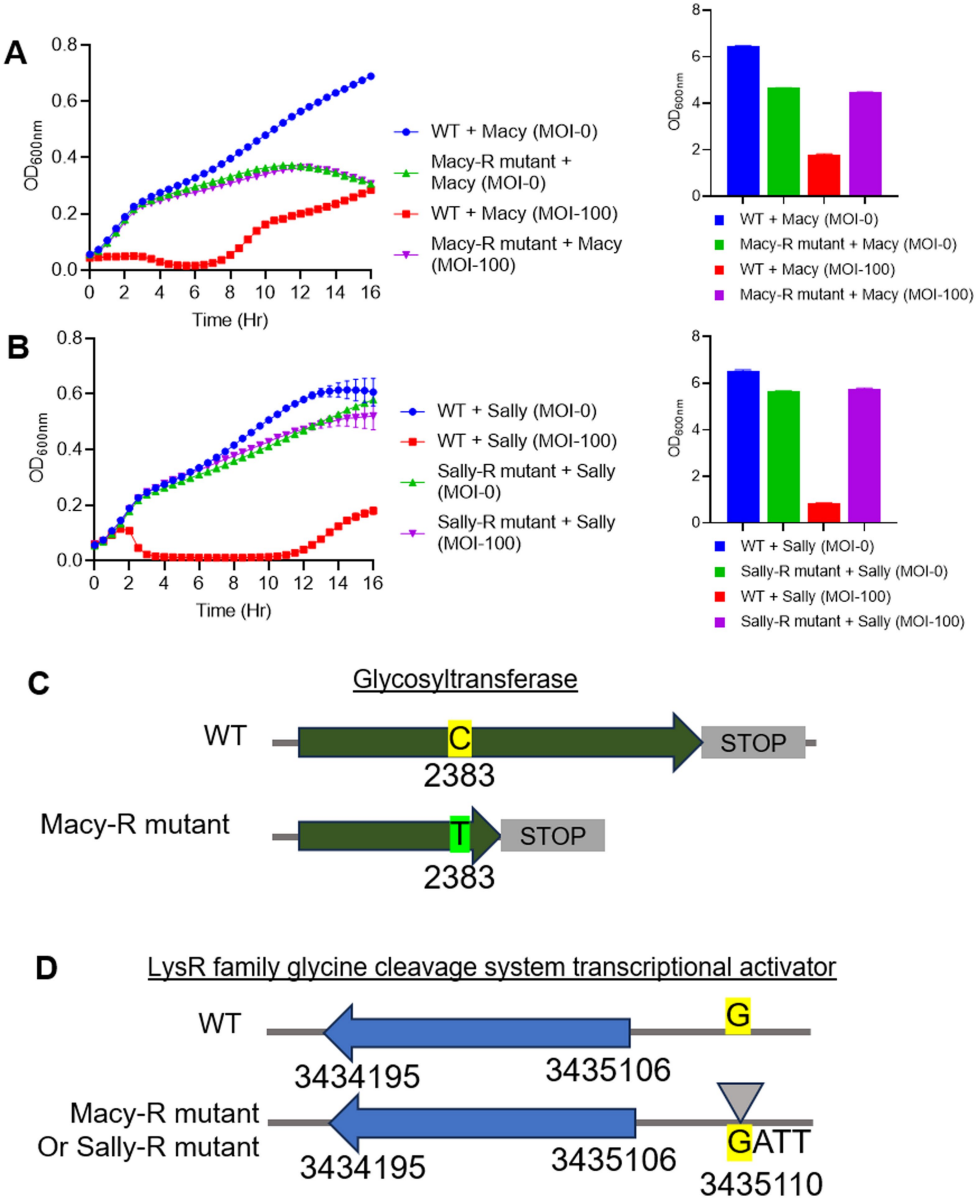
Identification of antiphage systems. Growth defects inImpaired growth of Macy **(A)** and Sally **(B)** resistant mutant of *Raoultella planticola*. **(C)** SNPs identified in the genome of Macy-resistant mutant. **(D)** SNPs identified in the genomes of Macy- and Sally-resistant mutants. and Sally-resistant mutant.

To evaluate the fitness costs associated with phage resistance, spontaneous resistant mutants of *R. planticola* strain RP8 were generated through repeated exposure to high phage titers. Growth dynamics of these mutants were compared with the wild type by monitoring OD₆₀₀ over 16 h using a microplate reader. The Macy-resistant RP8 variant exhibited a measurable fitness cost, showing significantly higher bacterial densities when infected with phage Macy at an MOI of 100 compared with the wild-type strain under identical conditions (Figure 10A). A similar pattern was observed for the Sally-resistant RP8 variant (Figure 10B). However, while no significant difference was detected between the Macy-resistant and wild-type strains during the first 7 h of infection, after which the resistant mutant declined, the Sally-resistant RP8 maintained growth comparable to wild-type–infected cultures throughout the experiment. These results indicate that resistance to Sally imposed a greater physiological burden than resistance to Macy.

Whole-genome sequencing of the resistant mutants revealed single nucleotide polymorphisms (SNPs) linked to potential anti-phage defense mechanisms (Figures 10C,D). In the Macy-resistant mutant, a premature stop codon (Q795*) was identified in a glycosyltransferase gene responsible for synthesizing and modifying surface polysaccharides, likely altering receptor structure and preventing phage adsorption. Both Macy- and Sally-resistant mutants also shared a GATT motif substitution at position G3435100 in the promoter region of a *LysR* family glycine-cleavage-system transcriptional activator gene. This promoter mutation may affect *LysR*-mediated regulation, reshaping metabolic or stress-response pathways involved in phage defense. Together, these findings suggest that *R. planticola* acquires phage resistance through mutations that alter surface architecture and regulatory networks, conferring protection at the expense of growth fitness.

## Discussion

Multidrug-resistant pathogens, particularly members of the ESKAPE pathogens (*Enterococcus faecium*, *Staphylococcus aureus*, *Klebsiella pneumoniae*, *Acinetobacter baumannii*, *Pseudomonas aeruginosa*, and *Enterobacter* species) represent the most formidable group of multidrug-resistant bacteria responsible for the majority of nosocomial infections worldwide (Dhungana et al., 2021). These organisms are known for their ability to “escape” the effects of existing antibiotics, prompting urgent calls for alternative therapeutic strategies. Although *Raoultella* species are not officially part of this group, their increasing isolation from clinical settings, particularly strains exhibiting carbapenem resistance such as *R. planticola*, suggests an emerging threat that mirrors the behavior of ESKAPE pathogens. The genetic and phenotypic similarity between *Raoultella* and *Klebsiella*, an established ESKAPE member, further underscores the potential for *Raoultella* to follow a similar trajectory in antimicrobial resistance and clinical relevance. As such, understanding and developing therapeutic options, including phage therapy, for drug-resistant *Raoultella* strains is a timely and important endeavor that aligns with global efforts to combat the growing challenge of antibiotic resistance.

In this study, we report the isolation and characterization of two virulent *Raoultella* phages, Macy and Sally, which display complementary features. Sally displayed a broad host range, infecting both environmental and clinical isolates of *R. ornithinolytica* and *R. planticola*, a characteristic shared with other cross-species *Raoultella* phages (Zamani et al., 2019; Kang et al., 2025; Fofanov et al., 2019). Such versatility may enhance therapeutic value in infections involving multiple strains. Macy, by contrast, was more specialized, infecting only *R. planticola*, but demonstrated exceptional replication parameters, including a remarkably high burst size (~8,375 PFU per cell) and very rapid adsorption (>99% within 2 min). This burst size is among the largest reported for bacteriophages, surpassed only by *Hafnia* phage Ca (Pan et al., 2022). The combination of high replication and rapid adsorption suggests Macy can efficiently suppress bacterial populations while reducing opportunities for resistance emergence.

An additional therapeutic feature of Macy is its encoded EPS depolymerase, consistent with the halo formation observed on host lawns. EPS depolymerases degrade extracellular polysaccharides in capsules or biofilms, facilitating phage access to bacterial cells and enhancing infection efficiency in both planktonic and biofilm-associated states (Chen et al., 2022; Vukotic et al., 2020; Wang et al., 2020). This activity is particularly valuable against *Raoultella* biofilms, which exhibit increased antibiotic tolerance and persistence in clinical and environmental settings (Hall-Stoodley et al., 2004; Hoiby et al., 2010; Donlan, 2000; Andrés-Lasheras et al., 2024). By breaking down the polysaccharide matrix, Macy can penetrate deeper biofilm layers and lyse otherwise protected bacterial cells, supporting its potential as a potent therapeutic agent. Although Sally lacks an identifiable depolymerase, its broader host range provides a complementary advantage for potential treatment applications.

Genomic analyses highlighted distinct evolutionary trajectories. Macy, a 148-kb dsDNA phage with ~45% GC content, displays a mosaic genome typical of T4-like viruses, reflecting frequent recombination and modular exchange (Grose and Casjens, 2014). Phylogenetically, it clustered within the *Klebsiella* II clade alongside phage Ro1, indicating shared ancestry with clinically relevant phages (Adriaenssens and Brister, 2017). The conservation of its lysis cassette, including i-spanin, o-spanin, and endolysin underscores strong purifying selection on structural and lytic modules (Cahill and Young, 2019; Young, 2013). In contrast, Sally has a smaller 48.5 kb genome (56% GC), aligning closely with its host genome and suggesting tighter co-adaptation. As a *Chivirus* (family Casjensviridae), it is more related to *Klebsiella* phages than other *Raoultella* phages. Despite overall genomic conservation, Sally encodes the largest number of unique genes in its proteomic cluster, highlighting its evolutionary distinctiveness.

Pangenome analysis further revealed divergent strategies, with the *Raoultella* phages (Macy and Ro1) encoding ~68% more strain-specific genes than related *Klebsiella* phages, suggesting stronger host-driven specialization. Only 27% of genes were shared across all examined genomes, consistent with high genomic plasticity in T4-like phages. By contrast, Sally and related *Chivirus* phages showed smaller genomes, higher conservation, and fewer strain-specific genes. These differences point to *Raoultella* phages as an underexplored but diverse evolutionary group.

Finally, mutations conferring resistance to Macy and Sally were primarily associated with alterations in surface glycosyltransferase genes and regulatory elements, consistent with mechanisms reported in other Gram-negative bacteria where receptor modification serves as a dominant antiphage strategy (Washizaki et al., 2016). The identified LysR-promoter mutation suggests that transcriptional reprogramming may complement structural receptor changes, reflecting a multi-layered defense system analogous to metabolically driven antiphage responses observed in *Pseudomonas* and *Escherichia coli* (Liang et al., 2025; Fitzpatrick et al., 2025). Although such adaptations effectively block phage infection, they often impose measurable fitness costs, reinforcing the evolutionary trade-offs that maintain phage–host coexistence in natural environments (Liang et al., 2025).

A key limitation of this study is the absence of *in vivo* validation, which is critical to assess therapeutic efficacy, stability, and ecological interactions under physiological conditions. Ongoing efforts, including evolutionary phage training, *in vivo* infection modeling, and phage pharmacokinetic studies, aim to address this gap and provide the necessary evidence for translational development.

## Conclusion

This study identifies two novel bacteriophages, Macy and Sally, as promising candidates for phage therapy against multidrug-resistant *R. planticola*. Their complementary biological properties, including Macy’s rapid replication and depolymerase activity and Sally’s broad host range and strong anti-biofilm capabilities, highlight their therapeutic potential. Together with genomic insights into phage resistance mechanisms, these findings advance the foundational knowledge required to develop targeted, evolution-informed phage cocktails. Future studies will expand therapeutic evaluations across additional clinical isolates and progress toward translational applications, ultimately supporting the development of effective alternative treatments for carbapenem-resistant *Raoultella* infections.

## Data Availability

The complete genome sequences of Macy and Sally are available in GenBank under accession numbers PX290106 and PX290107, respectively.

## Footnotes

1
https://www.bioinformatics.babraham.ac.uk/projects/fastqc/

2
hannonlab.cshl.edu

3
https://bioinfogp.cnb.csic.es/tools/venny/

4
https://github.com/tseemann/snippy
