# Supplementary material for: Two virulent bacteriophages targeting carbapenem-resistant Raoultella planticola
Source: Front Microbiol. 2026 Jan 9;16:1726803. doi: 10.3389/fmicb.2025.1726803 (PMC12827599; doi:10.3389/fmicb.2025.1726803)
Supplement: Supplementary file 1 [file Table_1.DOCX]

**Table S1.** General, physiological and structural characteristics of phages Macy and Sally

| **Feature** | **Macy** | **Sally** |
| --- | --- | --- |
| Capsid width (nm) | 55.70 | 99.4 |
| Tail length (nm) | 78.60 | 265.8 |
| Adsorption in 2 min (%) | 99 | 50 |
| Latent period (min) | 20 | 20 |
| Burst time (min) | 15 | 30 |
| Burst size | 8375 | 226 |

**Table S2:** Functional prediction of the *Raoultella* phage Macy, with supporting evidence

| **Gene** | **Start** | **Stop** |  | **Frame** | **Annotation** | **Category** | **mmseqs_top_hit** | **mmseqs_eVal** | **mmseqs_alnScore** | **mmseqs_seqIdentity** |
| --- | --- | --- | --- | --- | --- | --- | --- | --- | --- | --- |
| MACY_001 | 1876 | 2 |  | - | RIIA lysis inhibitor | lysis | NC_019400_p1 | 7.01E-201 | 629 | 0.543 |
| MACY_002 | 2053 | 1886 |  | - | hypothetical protein | unknown function | NC_019400_p2 | 3.77E-10 | 46 | 0.427 |
| MACY_003 | 2673 | 2041 |  | - | HNH endonuclease | DNA, RNA and nucleotide metabolism | NC_020843_p22 | 8.70E-42 | 145 | 0.478 |
| MACY_004 | 3026 | 2670 |  | - | endolysin | lysis | p14862 VI_10155 | 3.46E-20 | 79 | 0.393 |
| MACY_005 | 3225 | 3109 |  | - | hypothetical protein | unknown function | No_PHROG | No_PHROG | No_PHROG | No_PHROG |
| MACY_006 | 3643 | 3227 |  | - | hypothetical protein | unknown function | KT070867_p96 | 9.23E-28 | 100 | 0.452 |
| MACY_007 | 3822 | 3643 |  | - | hypothetical protein | unknown function | p180627 VI_08261 | 4.16E-14 | 58 | 0.52 |
| MACY_008 | 3985 | 3797 |  | - | hypothetical protein | unknown function | NC_025830_p152 | 1.10E-29 | 102 | 0.822 |
| MACY_009 | 4971 | 3985 |  | - | RNA ligase | DNA, RNA and nucleotide metabolism | NC_028887_p254 | 1.58E-46 | 164 | 0.382 |
| MACY_010 | 5245 | 5012 |  | - | hypothetical protein | unknown function | NC_029072_p149 | 9.17E-28 | 97 | 0.862 |
| MACY_011 | 5447 | 5238 |  | - | hypothetical protein | unknown function | KY555144_p130 | 1.60E-31 | 109 | 0.776 |
| MACY_012 | 5632 | 5444 |  | - | membrane protein | moron, auxiliary metabolic gene and host takeover | NC_022968_p261 | 7.83E-33 | 111 | 0.849 |
| MACY_013 | 5802 | 5629 |  | - | hypothetical protein | unknown function | NC_025830_p155 | 6.40E-35 | 117 | 1 |
| MACY_014 | 6296 | 5802 |  | - | phosphatase | other | MF490236_p17 | 1.34E-24 | 93 | 0.372 |
| MACY_015 | 6802 | 6293 |  | - | hypothetical protein | unknown function | KX431560_p264 | 7.38E-61 | 199 | 0.579 |
| MACY_016 | 6972 | 6802 |  | - | exonuclease | DNA, RNA and nucleotide metabolism | NC_025830_p157 | 5.69E-38 | 126 | 1 |
| MACY_017 | 7145 | 6969 |  | - | baseplate wedge subunit | tail | NC_022968_p258 | 3.52E-41 | 135 | 1 |
| MACY_018 | 7456 | 7145 |  | - | hypothetical protein | unknown function | NC_028659_p234 | 6.11E-69 | 218 | 0.99 |
| MACY_019 | 7696 | 7460 |  | - | hypothetical protein | unknown function | NC_028659_p233 | 7.43E-45 | 151 | 0.977 |
| MACY_020 | 7989 | 7690 |  | - | hypothetical protein | unknown function | NC_019400_p19 | 5.72E-71 | 223 | 1 |
| MACY_021 | 8135 | 7986 |  | - | hypothetical protein | unknown function | NC_019400_p20 | 1.20E-17 | 67 | 0.699 |
| MACY_022 | 8478 | 8140 |  | - | hypothetical protein | unknown function | NC_019400_p22 | 2.16E-69 | 220 | 0.919 |
| MACY_023 | 9736 | 8540 |  | - | ATPase | other | NC_019526_p53 | 4.38E-93 | 304 | 0.436 |
| MACY_024 | 10168 | 9800 |  | - | hypothetical protein | unknown function | KT001917_p6 | 2.16E-76 | 241 | 0.919 |
| MACY_025 | 10732 | 10223 |  | - | metal-dependent phosphohydrolase | other | KR296694_p108 | 2.72E-59 | 194 | 0.601 |
| MACY_026 | 11718 | 10696 |  | - | thymidylate kinase | other | NC_027351_p109 | 1.93E-146 | 455 | 0.673 |
| MACY_027 | 11845 | 12720 |  | + | HNH endonuclease | DNA, RNA and nucleotide metabolism | NC_005083_p40 | 2.79E-55 | 186 | 0.467 |
| MACY_028 | 12720 | 12980 |  | + | tail protein | tail | KR091942_p4 | 3.91E-54 | 174 | 0.972 |
| MACY_029 | 13030 | 13656 |  | + | HNH endonuclease | DNA, RNA and nucleotide metabolism | NC_020843_p22 | 4.64E-48 | 163 | 0.496 |
| MACY_030 | 13689 | 15095 |  | + | HNH endonuclease | DNA, RNA and nucleotide metabolism | KU885989_p28 | 5.90E-82 | 272 | 0.378 |
| MACY_031 | 15079 | 15489 |  | + | tail protein | tail | KR091942_p2 | 1.82E-48 | 160 | 0.711 |
| MACY_032 | 15596 | 15453 |  | - | hypothetical protein | unknown function | No_PHROG | No_PHROG | No_PHROG | No_PHROG |
| MACY_033 | 15814 | 15701 |  | - | hypothetical protein | unknown function | No_PHROG | No_PHROG | No_PHROG | No_PHROG |
| MACY_034 | 16037 | 16168 |  | + | hypothetical protein | unknown function | No_PHROG | No_PHROG | No_PHROG | No_PHROG |
| MACY_035 | 16146 | 16406 |  | + | hypothetical protein | unknown function | NC_023717_p129 | 5.62E-22 | 81 | 0.538 |
| MACY_036 | 16433 | 17077 |  | + | hypothetical protein | unknown function | MG250486_p33 | 1.36E-121 | 376 | 0.852 |
| MACY_037 | 17151 | 17486 |  | + | MazG-like pyrophosphatase | other | MG603697_p4 | 4.48E-23 | 86 | 0.462 |
| MACY_038 | 17483 | 17833 |  | + | hypothetical protein | unknown function | NC_022323_p20 | 5.49E-65 | 209 | 0.868 |
| MACY_039 | 17826 | 18236 |  | + | hypothetical protein | unknown function | MG250486_p36 | 7.57E-81 | 254 | 0.889 |
| MACY_040 | 18233 | 18391 |  | + | hypothetical protein | unknown function | NC_025830_p23 | 1.84E-30 | 104 | 1 |
| MACY_041 | 18518 | 19360 |  | + | hypothetical protein | unknown function | KY652726_p206 | 2.04E-147 | 454 | 0.814 |
| MACY_042 | 19414 | 19506 |  | + | hypothetical protein | unknown function | No_PHROG | No_PHROG | No_PHROG | No_PHROG |
| MACY_043 | 19503 | 19598 |  | + | hypothetical protein | unknown function | No_PHROG | No_PHROG | No_PHROG | No_PHROG |
| MACY_044 | 19643 | 19858 |  | + | hypothetical protein | unknown function | NC_016071_p202 | 1.68E-15 | 64 | 0.582 |
| MACY_045 | 19900 | 20034 |  | + | hypothetical protein | unknown function | No_PHROG | No_PHROG | No_PHROG | No_PHROG |
| MACY_046 | 20162 | 20025 |  | - | hypothetical protein | unknown function | No_PHROG | No_PHROG | No_PHROG | No_PHROG |
| MACY_047 | 20335 | 20183 |  | - | hypothetical protein | unknown function | KY652726_p204 | 1.50E-25 | 91 | 0.85 |
| MACY_048 | 20303 | 20725 |  | + | hypothetical protein | unknown function | NC_019400_p55 | 9.44E-12 | 51 | 0.451 |
| MACY_049 | 20764 | 20931 |  | + | hypothetical protein | unknown function | KU522583_p8 | 3.56E-31 | 106 | 0.935 |
| MACY_050 | 21025 | 21810 |  | + | hypothetical protein | unknown function | NC_030910_p154 | 3.92E-34 | 124 | 0.436 |
| MACY_051 | 21785 | 21955 |  | + | hypothetical protein | unknown function | No_PHROG | No_PHROG | No_PHROG | No_PHROG |
| MACY_052 | 21971 | 22237 |  | + | hypothetical protein | unknown function | No_PHROG | No_PHROG | No_PHROG | No_PHROG |
| MACY_053 | 22234 | 22392 |  | + | hypothetical protein | unknown function | No_PHROG | No_PHROG | No_PHROG | No_PHROG |
| MACY_054 | 22474 | 22989 |  | + | hypothetical protein | unknown function | NC_028659_p205 | 1.98E-90 | 284 | 0.802 |
| MACY_055 | 23049 | 23432 |  | + | hypothetical protein | unknown function | KR052480_p255 | 2.48E-62 | 201 | 0.723 |
| MACY_056 | 23401 | 23796 |  | + | hypothetical protein | unknown function | MF979563_p140 | 5.48E-46 | 152 | 0.744 |
| MACY_057 | 23771 | 23986 |  | + | hypothetical protein | unknown function | KR698074_p46 | 2.25E-39 | 130 | 0.914 |
| MACY_058 | 23979 | 24080 |  | + | hypothetical protein | unknown function | No_PHROG | No_PHROG | No_PHROG | No_PHROG |
| MACY_059 | 24320 | 24604 |  | + | hypothetical protein | unknown function | NC_027351_p133 | 1.06E-75 | 237 | 1 |
| MACY_060 | 24642 | 24860 |  | + | hypothetical protein | unknown function | NC_027351_p134 | 2.54E-51 | 165 | 1 |
| MACY_061 | 24871 | 25074 |  | + | hypothetical protein | unknown function | MF979560_p142 | 3.42E-50 | 163 | 1 |
| MACY_062 | 25145 | 25468 |  | + | hypothetical protein | unknown function | NC_022968_p212 | 3.23E-63 | 202 | 0.895 |
| MACY_063 | 25576 | 26346 |  | + | hypothetical protein | unknown function | KX961385_p40 | 4.89E-68 | 226 | 0.45 |
| MACY_064 | 26396 | 26545 |  | + | hypothetical protein | unknown function | No_PHROG | No_PHROG | No_PHROG | No_PHROG |
| MACY_065 | 26542 | 27036 |  | + | hypothetical protein | unknown function | NC_017974_p149 | 1.86E-81 | 257 | 0.796 |
| MACY_066 | 27067 | 27354 |  | + | hypothetical protein | unknown function | No_PHROG | No_PHROG | No_PHROG | No_PHROG |
| MACY_067 | 27503 | 27351 |  | - | hypothetical protein | unknown function | No_PHROG | No_PHROG | No_PHROG | No_PHROG |
| MACY_068 | 27670 | 27539 |  | - | hypothetical protein | unknown function | No_PHROG | No_PHROG | No_PHROG | No_PHROG |
| MACY_069 | 27802 | 28161 |  | + | hypothetical protein | unknown function | NC_031934_p206 | 2.84E-42 | 141 | 0.631 |
| MACY_070 | 28236 | 28565 |  | + | hypothetical protein | unknown function | NC_017974_p156 | 2.38E-65 | 207 | 0.932 |
| MACY_071 | 28614 | 28844 |  | + | hypothetical protein | unknown function | NC_028659_p189 | 2.39E-48 | 157 | 0.966 |
| MACY_072 | 28844 | 29023 |  | + | hypothetical protein | unknown function | No_PHROG | No_PHROG | No_PHROG | No_PHROG |
| MACY_073 | 29033 | 29353 |  | + | hypothetical protein | unknown function | No_PHROG | No_PHROG | No_PHROG | No_PHROG |
| MACY_074 | 29347 | 29580 |  | + | hypothetical protein | unknown function | No_PHROG | No_PHROG | No_PHROG | No_PHROG |
| MACY_075 | 29757 | 29650 |  | - | hypothetical protein | unknown function | No_PHROG | No_PHROG | No_PHROG | No_PHROG |
| MACY_076 | 29929 | 29840 |  | - | hypothetical protein | unknown function | No_PHROG | No_PHROG | No_PHROG | No_PHROG |
| MACY_077 | 30059 | 29919 |  | - | hypothetical protein | unknown function | No_PHROG | No_PHROG | No_PHROG | No_PHROG |
| MACY_078 | 30221 | 30120 |  | - | hypothetical protein | unknown function | No_PHROG | No_PHROG | No_PHROG | No_PHROG |
| MACY_079 | 30304 | 30444 |  | + | hypothetical protein | unknown function | No_PHROG | No_PHROG | No_PHROG | No_PHROG |
| MACY_080 | 30471 | 30650 |  | + | hypothetical protein | unknown function | No_PHROG | No_PHROG | No_PHROG | No_PHROG |
| MACY_081 | 30714 | 30884 |  | + | hypothetical protein | unknown function | No_PHROG | No_PHROG | No_PHROG | No_PHROG |
| MACY_082 | 31025 | 31171 |  | + | hypothetical protein | unknown function | No_PHROG | No_PHROG | No_PHROG | No_PHROG |
| MACY_083 | 31149 | 31361 |  | + | hypothetical protein | unknown function | No_PHROG | No_PHROG | No_PHROG | No_PHROG |
| MACY_084 | 31380 | 31469 |  | + | hypothetical protein | unknown function | No_PHROG | No_PHROG | No_PHROG | No_PHROG |
| MACY_085 | 31814 | 31527 |  | - | hypothetical protein | unknown function | KY652726_p179 | 2.04E-36 | 124 | 0.663 |
| MACY_086 | 31997 | 31863 |  | - | hypothetical protein | unknown function | No_PHROG | No_PHROG | No_PHROG | No_PHROG |
| MACY_087 | 32371 | 32108 |  | - | hypothetical protein | unknown function | No_PHROG | No_PHROG | No_PHROG | No_PHROG |
| MACY_088 | 32727 | 32371 |  | - | hypothetical protein | unknown function | MG250486_p57 | 6.97E-54 | 172 | 1 |
| MACY_089 | 32922 | 32788 |  | - | hypothetical protein | unknown function | No_PHROG | No_PHROG | No_PHROG | No_PHROG |
| MACY_090 | 33014 | 32901 |  | - | hypothetical protein | unknown function | NC_028933_p7 | 1.13E-07 | 40 | 0.672 |
| MACY_091 | 33205 | 33011 |  | - | hypothetical protein | unknown function | No_PHROG | No_PHROG | No_PHROG | No_PHROG |
| MACY_092 | 33347 | 33180 |  | - | hypothetical protein | unknown function | NC_019400_p92 | 7.88E-13 | 54 | 0.644 |
| MACY_093 | 33539 | 33354 |  | - | hypothetical protein | unknown function | No_PHROG | No_PHROG | No_PHROG | No_PHROG |
| MACY_094 | 33836 | 33576 |  | - | hypothetical protein | unknown function | NC_028659_p177 | 1.16E-33 | 115 | 0.659 |
| MACY_095 | 34015 | 33833 |  | - | hypothetical protein | unknown function | NC_028659_p176 | 6.08E-35 | 117 | 0.916 |
| MACY_096 | 34175 | 34020 |  | - | hypothetical protein | unknown function | NC_028659_p175 | 1.78E-41 | 136 | 1 |
| MACY_097 | 34548 | 34228 |  | - | hypothetical protein | unknown function | No_PHROG | No_PHROG | No_PHROG | No_PHROG |
| MACY_098 | 34787 | 34548 |  | - | hypothetical protein | unknown function | NC_028659_p174 | 9.52E-62 | 196 | 1 |
| MACY_099 | 35178 | 34774 |  | - | hypothetical protein | unknown function | NC_029000_p10 | 1.86E-50 | 165 | 0.786 |
| MACY_100 | 35340 | 35182 |  | - | hypothetical protein | unknown function | NC_030941_p36 | 8.72E-25 | 88 | 0.799 |
| MACY_101 | 35516 | 35349 |  | - | hypothetical protein | unknown function | KY652726_p160 | 4.33E-34 | 115 | 0.969 |
| MACY_102 | 35758 | 35513 |  | - | hypothetical protein | unknown function | NC_028659_p169 | 2.37E-45 | 149 | 0.889 |
| MACY_103 | 36036 | 35755 |  | - | hypothetical protein | unknown function | MG250486_p67 | 1.48E-39 | 132 | 0.766 |
| MACY_104 | 36178 | 36029 |  | - | hypothetical protein | unknown function | NC_019400_p20 | 2.05E-11 | 49 | 0.669 |
| MACY_105 | 36378 | 36175 |  | - | hypothetical protein | unknown function | NC_019400_p103 | 2.28E-33 | 113 | 0.817 |
| MACY_106 | 36411 | 37622 |  | + | endonuclease | DNA, RNA and nucleotide metabolism | KY683735_p127 | 2.09E-45 | 162 | 0.316 |
| MACY_107 | 37631 | 37882 |  | + | hypothetical protein | unknown function | KY652726_p154 | 1.15E-56 | 181 | 1 |
| MACY_108 | 37836 | 38159 |  | + | hypothetical protein | unknown function | KY652726_p153 | 1.18E-67 | 214 | 1 |
| MACY_109 | 38560 | 38186 |  | - | endonuclease VII | DNA, RNA and nucleotide metabolism | No_PHROG | No_PHROG | No_PHROG | No_PHROG |
| MACY_110 | 39052 | 38573 |  | - | virion structural protein | head and packaging | NC_028248_p181 | 1.51E-85 | 269 | 0.847 |
| MACY_111 | 39355 | 39125 |  | - | hypothetical protein | unknown function | NC_016071_p169 | 6.63E-53 | 170 | 1 |
| MACY_112 | 39528 | 39352 |  | - | hypothetical protein | unknown function | KT001917_p120 | 4.37E-38 | 126 | 1 |
| MACY_113 | 39727 | 39515 |  | - | hypothetical protein | unknown function | NC_028659_p160 | 9.95E-46 | 149 | 0.992 |
| MACY_114 | 40794 | 39724 |  | - | nucleotidyltransferase | DNA, RNA and nucleotide metabolism | NC_031245_p308 | 1.49E-41 | 147 | 0.427 |
| MACY_115 | 41178 | 40804 |  | - | hypothetical protein | unknown function | NC_022968_p162 | 2.47E-67 | 214 | 0.827 |
| MACY_116 | 41671 | 41180 |  | - | hydrolase | other | NC_028672_p36 | 2.32E-64 | 207 | 0.663 |
| MACY_117 | 41886 | 41671 |  | - | membrane protein | moron, auxiliary metabolic gene and host takeover | NC_016071_p161 | 2.88E-34 | 116 | 0.783 |
| MACY_118 | 42314 | 41883 |  | - | hypothetical protein | unknown function | KY652726_p143 | 4.09E-64 | 204 | 0.933 |
| MACY_119 | 42742 | 42266 |  | - | HNH endonuclease | DNA, RNA and nucleotide metabolism | KY385423_p40 | 2.34E-25 | 95 | 0.4 |
| MACY_120 | 42942 | 42742 |  | - | hypothetical protein | unknown function | MF979563_p51 | 5.40E-42 | 138 | 0.977 |
| MACY_121 | 43394 | 42942 |  | - | hypothetical protein | unknown function | KY652726_p141 | 6.81E-80 | 252 | 0.825 |
| MACY_122 | 44638 | 43391 |  | - | tRNA nucleotidyltransferase | DNA, RNA and nucleotide metabolism | NC_023719_p514 | 2.03E-113 | 363 | 0.519 |
| MACY_123 | 44914 | 44648 |  | - | hypothetical protein | unknown function | NC_019400_p125 | 1.41E-46 | 153 | 0.827 |
| MACY_124 | 45203 | 44919 |  | - | hypothetical protein | unknown function | NC_031047_p3 | 1.66E-44 | 147 | 0.756 |
| MACY_125 | 45517 | 45200 |  | - | hypothetical protein | unknown function | NC_019517_p118 | 6.80E-44 | 147 | 0.635 |
| MACY_126 | 45758 | 45510 |  | - | hypothetical protein | unknown function | NC_018275_p33 | 4.22E-46 | 151 | 0.798 |
| MACY_127 | 45922 | 45755 |  | - | hypothetical protein | unknown function | NC_019400_p128 | 2.27E-37 | 124 | 1 |
| MACY_128 | 46118 | 45897 |  | - | hypothetical protein | unknown function | NC_027351_p172 | 1.46E-39 | 131 | 0.907 |
| MACY_129 | 46387 | 46118 |  | - | hypothetical protein | unknown function | NC_019517_p117 | 7.20E-37 | 124 | 0.726 |
| MACY_130 | 46620 | 46471 |  | - | hypothetical protein | unknown function | NC_022968_p146 | 8.20E-34 | 113 | 1 |
| MACY_131 | 47266 | 46583 |  | - | DNA methyltransferase | other | NC_011103_p96 | 2.68E-45 | 156 | 0.476 |
| MACY_132 | 48180 | 47266 |  | - | ATP-dependent protease | other | NC_017974_p205 | 3.21E-135 | 421 | 0.711 |
| MACY_133 | 48664 | 48170 |  | - | hypothetical protein | unknown function | MG250486_p91 | 2.02E-100 | 312 | 0.905 |
| MACY_134 | 48860 | 48672 |  | - | hypothetical protein | unknown function | NC_028248_p170 | 3.37E-26 | 95 | 0.725 |
| MACY_135 | 49542 | 48961 |  | - | DprA-like DNA recombination-mediator protein | DNA, RNA and nucleotide metabolism | NC_031043_p89 | 5.24E-42 | 145 | 0.493 |
| MACY_136 | 50204 | 49599 |  | - | endonuclease | DNA, RNA and nucleotide metabolism | KU204984_p45 | 3.53E-18 | 74 | 0.387 |
| MACY_137 | 51002 | 50214 |  | - | PhoH-like phosphate starvation-inducible | other | KT995480_p45 | 1.94E-56 | 189 | 0.53 |
| MACY_138 | 51567 | 51013 |  | - | endolysin | lysis | p199731 VI_00081 | 1.50E-59 | 196 | 0.556 |
| MACY_139 | 51966 | 51586 |  | - | hypothetical protein | unknown function | KT001917_p107 | 2.98E-64 | 205 | 0.871 |
| MACY_140 | 52106 | 51966 |  | - | hypothetical protein | unknown function | KT001917_p106 | 1.41E-20 | 75 | 0.932 |
| MACY_141 | 52461 | 52186 |  | - | thioredoxin domain | DNA, RNA and nucleotide metabolism | KX925554_p168 | 2.79E-06 | 38 | 0.321 |
| MACY_142 | 52805 | 52464 |  | - | virion structural protein | head and packaging | NC_027351_p187 | 1.81E-59 | 191 | 0.813 |
| MACY_143 | 53903 | 52815 |  | - | ribonucleoside diphosphate reductase small subunit | DNA, RNA and nucleotide metabolism | KT995480_p118 | 7.71E-73 | 243 | 0.415 |
| MACY_144 | 56139 | 53941 |  | - | ribonucleotide reductase large subunit | DNA, RNA and nucleotide metabolism | KJ019113_p163 | 9.09E-150 | 479 | 0.469 |
| MACY_145 | 56493 | 56263 |  | - | hypothetical protein | unknown function | NC_016071_p136 | 7.36E-50 | 161 | 0.99 |
| MACY_146 | 56969 | 56490 |  | - | hypothetical protein | unknown function | NC_028672_p55 | 8.72E-92 | 287 | 0.859 |
| MACY_147 | 57492 | 56962 |  | - | HNH endonuclease | DNA, RNA and nucleotide metabolism | NC_019516_p145 | 6.47E-32 | 112 | 0.507 |
| MACY_148 | 58357 | 57479 |  | - | thymidylate synthase | DNA, RNA and nucleotide metabolism | NC_008198_p65 | 1.46E-19 | 84 | 0.319 |
| MACY_149 | 58775 | 58458 |  | - | hypothetical protein | unknown function | NC_022968_p129 | 1.17E-62 | 200 | 0.894 |
| MACY_150 | 58972 | 58772 |  | - | hypothetical protein | unknown function | NC_019517_p99 | 4.76E-36 | 121 | 0.877 |
| MACY_151 | 59586 | 58972 |  | - | unknown function | unknown function | AP018399_p172 | 6.67E-23 | 89 | 0.338 |
| MACY_152 | 60172 | 59567 |  | - | deoxynucleoside monophosphate kinase | other | KX507046_p83 | 9.03E-30 | 111 | 0.341 |
| MACY_153 | 60351 | 60172 |  | - | hypothetical protein | unknown function | KT001917_p94 | 1.45E-15 | 62 | 0.523 |
| MACY_154 | 61353 | 60379 |  | - | DNA polymerase exonuclease subunit | DNA, RNA and nucleotide metabolism | KU160647_p41 | 3.69E-87 | 280 | 0.565 |
| MACY_155 | 61556 | 61353 |  | - | hypothetical protein | unknown function | KT001917_p90 | 3.76E-34 | 116 | 0.855 |
| MACY_156 | 62031 | 61546 |  | - | endonuclease VII | DNA, RNA and nucleotide metabolism | NC_028672_p63 | 2.30E-49 | 164 | 0.579 |
| MACY_157 | 62687 | 62076 |  | - | RuvC-like Holliday junction resolvase | DNA, RNA and nucleotide metabolism | p35032 VI_04131 | 1.18E-13 | 62 | 0.28 |
| MACY_158 | 62988 | 62668 |  | - | hypothetical protein | unknown function | NC_022323_p89 | 1.08E-63 | 203 | 0.907 |
| MACY_159 | 63884 | 62985 |  | - | exonuclease | DNA, RNA and nucleotide metabolism | NC_019402_p42 | 3.11E-40 | 145 | 0.375 |
| MACY_160 | 64039 | 63881 |  | - | hypothetical protein | unknown function | No_PHROG | No_PHROG | No_PHROG | No_PHROG |
| MACY_161 | 64612 | 64124 |  | - | HNH endonuclease | DNA, RNA and nucleotide metabolism | p180900 VI_03586 | 2.37E-37 | 127 | 0.627 |
| MACY_162 | 65096 | 64599 |  | - | HNH endonuclease | DNA, RNA and nucleotide metabolism | NC_024134_p84 | 6.90E-81 | 255 | 0.854 |
| MACY_163 | 65550 | 65044 |  | - | hypothetical protein | unknown function | p137170 VI_02792 | 3.06E-47 | 161 | 0.509 |
| MACY_164 | 65945 | 65550 |  | - | hypothetical protein | unknown function | NC_020079_p128 | 8.47E-43 | 143 | 0.616 |
| MACY_165 | 66099 | 65905 |  | - | hypothetical protein | unknown function | NC_023717_p255 | 8.88E-27 | 94 | 0.776 |
| MACY_166 | 67052 | 66162 |  | - | lipoprotein | other | p13345 VI_10141 | 9.84E-43 | 151 | 0.345 |
| MACY_167 | 67209 | 67045 |  | - | hypothetical protein | unknown function | NC_022968_p115 | 2.22E-38 | 127 | 1 |
| MACY_168 | 67409 | 67209 |  | - | membrane protein | moron, auxiliary metabolic gene and host takeover | NC_022968_p114 | 9.50E-27 | 94 | 0.72 |
| MACY_169 | 67771 | 67409 |  | - | transcriptional regulator | transcription regulation | NC_023568_p222 | 6.85E-21 | 79 | 0.458 |
| MACY_170 | 68093 | 67839 |  | - | hypothetical protein | unknown function | NC_016071_p113 | 1.96E-44 | 146 | 0.884 |
| MACY_171 | 68793 | 68116 |  | - | nucleotide pyrophosphohydrolase | other | NC_029057_p89 | 1.70E-34 | 125 | 0.356 |
| MACY_172 | 69056 | 68802 |  | - | hypothetical protein | unknown function | NC_028659_p111 | 8.09E-68 | 213 | 1 |
| MACY_173 | 69393 | 69043 |  | - | hypothetical protein | unknown function | NC_028659_p110 | 1.10E-60 | 195 | 0.808 |
| MACY_174 | 69853 | 69395 |  | - | hypothetical protein | unknown function | p167551 VI_05449 | 5.43E-21 | 79 | 0.471 |
| MACY_175 | 70467 | 69853 |  | - | HNH endonuclease | DNA, RNA and nucleotide metabolism | NC_020843_p22 | 2.25E-16 | 71 | 0.334 |
| MACY_176 | 71756 | 70449 |  | - | ATP-dependent DNA ligase | DNA, RNA and nucleotide metabolism | NC_019913_p32 | 2.06E-79 | 263 | 0.414 |
| MACY_177 | 72073 | 71756 |  | - | hypothetical protein | unknown function | No_PHROG | No_PHROG | No_PHROG | No_PHROG |
| MACY_178 | 72849 | 72073 |  | - | Sir2 (NAD-dependent deacetylase) | DNA, RNA and nucleotide metabolism | KY630187_p112 | 1.04E-105 | 333 | 0.702 |
| MACY_179 | 73266 | 72859 |  | - | ATPase | other | KX552041_p115 | 1.78E-32 | 115 | 0.466 |
| MACY_180 | 73610 | 73266 |  | - | hypothetical protein | unknown function | MF347637_p136 | 1.67E-34 | 119 | 0.567 |
| MACY_181 | 74569 | 73607 |  | - | RNA ligase and tail fiber protein attachment catalyst | tail | MF403008_p261 | 2.26E-54 | 190 | 0.355 |
| MACY_182 | 74735 | 74538 |  | - | hypothetical protein | unknown function | KY652726_p83 | 2.74E-45 | 147 | 1 |
| MACY_183 | 75075 | 74719 |  | - | hypothetical protein | unknown function | NC_019401_p176 | 2.09E-62 | 200 | 0.808 |
| MACY_184 | 75670 | 75077 |  | - | phosphoesterase | other | NC_024792_p125 | 3.10E-43 | 149 | 0.462 |
| MACY_185 | 75906 | 75667 |  | - | hypothetical protein | unknown function | NC_022968_p99 | 1.53E-52 | 169 | 0.999 |
| MACY_186 | 76187 | 75903 |  | - | hypothetical protein | unknown function | NC_023693_p103 | 2.04E-66 | 211 | 1 |
| MACY_187 | 76453 | 76184 |  | - | hypothetical protein | unknown function | NC_019517_p79 | 9.26E-56 | 180 | 0.94 |
| MACY_188 | 76548 | 76450 |  | - | hypothetical protein | unknown function | No_PHROG | No_PHROG | No_PHROG | No_PHROG |
| MACY_189 | 76894 | 76571 |  | - | hypothetical protein | unknown function | KR698074_p204 | 3.12E-22 | 82 | 0.623 |
| MACY_190 | 77218 | 77592 |  | + | hypothetical protein | unknown function | NC_020201_p192 | 1.69E-68 | 218 | 0.898 |
| MACY_191 | 77589 | 78389 |  | + | ribose-phosphate pyrophosphokinase | other | NC_024383_p145 | 1.59E-83 | 271 | 0.528 |
| MACY_192 | 78451 | 80151 |  | + | nicotinamide phosphoribosyl transferase | other | NC_031034_p67 | 4.75E-198 | 612 | 0.584 |
| MACY_193 | 80287 | 80478 |  | + | hypothetical protein | unknown function | NC_022968_p90 | 2.48E-44 | 145 | 1 |
| MACY_194 | 80471 | 80683 |  | + | hypothetical protein | unknown function | KR698074_p147 | 5.21E-20 | 75 | 0.613 |
| MACY_195 | 80758 | 81003 |  | + | hypothetical protein | unknown function | NC_020201_p198 | 8.30E-51 | 164 | 0.952 |
| MACY_196 | 81067 | 81186 |  | + | hypothetical protein | unknown function | NC_016071_p90 | 2.95E-24 | 85 | 1 |
| MACY_197 | 81167 | 81361 |  | + | hypothetical protein | unknown function | NC_028672_p98 | 9.33E-35 | 117 | 0.911 |
| MACY_198 | 81625 | 81936 |  | + | hypothetical protein | unknown function | MG250486_p158 | 7.69E-71 | 223 | 1 |
| MACY_199 | 82134 | 82391 |  | + | hypothetical protein | unknown function | MG250486_p159 | 6.64E-31 | 106 | 0.811 |
| MACY_200 | 82388 | 82525 |  | + | membrane protein | moron, auxiliary metabolic gene and host takeover | MG250486_p160 | 3.21E-29 | 100 | 1 |
| MACY_201 | 82566 | 83177 |  | + | hypothetical protein | unknown function | MF988720_p180 | 6.04E-106 | 331 | 0.798 |
| MACY_202 | 83255 | 83902 |  | + | tail fiber protein | tail | KY554769_p95 | 3.41E-86 | 274 | 0.642 |
| MACY_203 | 84051 | 84200 |  | + | hypothetical protein | unknown function | No_PHROG | No_PHROG | No_PHROG | No_PHROG |
| MACY_204 | 84724 | 84476 |  | - | hypothetical protein | unknown function | No_PHROG | No_PHROG | No_PHROG | No_PHROG |
| MACY_205 | 85175 | 85525 |  | + | hypothetical protein | unknown function | KY883634_p52 | 2.41E-47 | 157 | 0.665 |
| MACY_206 | 85752 | 86315 |  | + | HNH endonuclease | DNA, RNA and nucleotide metabolism | MG812495_p46 | 2.10E-32 | 116 | 0.457 |
| MACY_207 | 86299 | 86397 |  | + | hypothetical protein | unknown function | No_PHROG | No_PHROG | No_PHROG | No_PHROG |
| MACY_208 | 86789 | 86983 |  | + | hypothetical protein | unknown function | No_PHROG | No_PHROG | No_PHROG | No_PHROG |
| MACY_209 | 87092 | 87238 |  | + | hypothetical protein | unknown function | No_PHROG | No_PHROG | No_PHROG | No_PHROG |
| MACY_210 | 87883 | 87671 |  | - | hypothetical protein | unknown function | No_PHROG | No_PHROG | No_PHROG | No_PHROG |
| MACY_211 | 88494 | 89951 |  | + | hypothetical protein | unknown function | NC_019400_p196 | 0 | 971 | 0.952 |
| MACY_212 | 89948 | 90352 |  | + | Rz-like spanin | lysis | NC_016071_p77 | 1.44E-61 | 198 | 0.73 |
| MACY_213 | 90349 | 90639 |  | + | Rz-like spanin | lysis | NC_012223_p6 | 4.71E-14 | 59 | 0.371 |
| MACY_214 | 90768 | 92252 |  | + | terminase large subunit | head and packaging | NC_025436_p94 | 1.91E-120 | 374 | 0.771 |
| MACY_215 | 92224 | 92337 |  | + | hypothetical protein | unknown function | No_PHROG | No_PHROG | No_PHROG | No_PHROG |
| MACY_216 | 92403 | 93809 |  | + | hypothetical protein | unknown function | KR296695_p7 | 4.47E-88 | 277 | 1 |
| MACY_217 | 93878 | 94504 |  | + | hypothetical protein | unknown function | KR296695_p9 | 1.02E-39 | 135 | 0.833 |
| MACY_218 | 94501 | 95499 |  | + | hypothetical protein | unknown function | MF805809_p114 | 1.72E-60 | 207 | 0.37 |
| MACY_219 | 95519 | 95941 |  | + | head decoration | head and packaging | KX507046_p113 | 8.52E-45 | 150 | 0.605 |
| MACY_220 | 95963 | 96976 |  | + | major head protein | head and packaging | p33806 VI_03880 | 2.06E-20 | 87 | 0.248 |
| MACY_221 | 97097 | 97615 |  | + | hypothetical protein | unknown function | NC_020843_p131 | 5.89E-54 | 180 | 0.539 |
| MACY_222 | 97635 | 98027 |  | + | head fiber protein | head and packaging | MG250486_p176 | 6.44E-50 | 163 | 0.628 |
| MACY_223 | 98447 | 98049 |  | - | membrane protein | moron, auxiliary metabolic gene and host takeover | MG250486_p177 | 2.04E-86 | 270 | 0.963 |
| MACY_224 | 98583 | 98458 |  | - | hypothetical protein | unknown function | No_PHROG | No_PHROG | No_PHROG | No_PHROG |
| MACY_225 | 98572 | 99090 |  | + | hypothetical protein | unknown function | KY883640_p18 | 1.07E-48 | 164 | 0.533 |
| MACY_226 | 99156 | 99629 |  | + | tail completion or Neck1 protein | connector | KX664695_p144 | 3.33E-24 | 92 | 0.357 |
| MACY_227 | 99626 | 100063 |  | + | minor head protein | head and packaging | NC_020079_p156 | 1.65E-60 | 196 | 0.735 |
| MACY_228 | 100060 | 100605 |  | + | hypothetical protein | unknown function | NC_028672_p116 | 4.28E-100 | 315 | 0.838 |
| MACY_229 | 100638 | 102134 |  | + | tail sheath | tail | NC_021067_p46 | 4.50E-69 | 236 | 0.343 |
| MACY_230 | 102138 | 102611 |  | + | virion structural protein | head and packaging | NC_019544_p9 | 1.48E-30 | 109 | 0.462 |
| MACY_231 | 102595 | 102696 |  | + | hypothetical protein | unknown function | No_PHROG | No_PHROG | No_PHROG | No_PHROG |
| MACY_232 | 102693 | 103172 |  | + | tail assembly chaperone | tail | NC_020079_p160 | 1.80E-67 | 217 | 0.72 |
| MACY_233 | 103295 | 103447 |  | + | hypothetical protein | unknown function | NC_023717_p18 | 1.95E-25 | 90 | 0.832 |
| MACY_234 | 103498 | 105903 |  | + | tail length tape measure protein | tail | NC_022323_p48 | 0 | 1084 | 0.7 |
| MACY_235 | 105985 | 106857 |  | + | hypothetical protein | unknown function | NC_024354_p21 | 1.08E-133 | 415 | 0.751 |
| MACY_236 | 106857 | 107207 |  | + | virion structural protein | head and packaging | NC_020204_p5 | 9.38E-11 | 50 | 0.463 |
| MACY_237 | 107210 | 108199 |  | + | baseplate hub | tail | NC_020843_p99 | 1.82E-36 | 135 | 0.286 |
| MACY_238 | 108199 | 108900 |  | + | baseplate spike | tail | KY883640_p56 | 2.61E-17 | 75 | 0.3 |
| MACY_239 | 108910 | 109524 |  | + | hypothetical protein | unknown function | KX552041_p157 | 1.23E-103 | 324 | 0.787 |
| MACY_240 | 109645 | 111693 |  | + | EPS depolymerase | tail | NC_031062_p87 | 1.25E-16 | 78 | 0.414 |
| MACY_241 | 111766 | 114063 |  | + | hypothetical protein | unknown function | KY652726_p27 | 1.05E-99 | 349 | 0.512 |
| MACY_242 | 114066 | 114911 |  | + | hypothetical protein | unknown function | No_PHROG | No_PHROG | No_PHROG | No_PHROG |
| MACY_243 | 114981 | 115817 |  | + | tail protein | tail | MG250486_p199 | 8.08E-194 | 588 | 1 |
| MACY_244 | 115821 | 117659 |  | + | tail fiber protein | tail | p125858 VI_11207 | 2.35E-21 | 91 | 0.241 |
| MACY_245 | 117746 | 119239 |  | + | baseplate wedge subunit | tail | p248291 VI_09499 | 5.55E-17 | 77 | 0.195 |
| MACY_246 | 119248 | 119877 |  | + | baseplate protein | tail | NC_024134_p34 | 7.78E-116 | 360 | 0.831 |
| MACY_247 | 119887 | 120933 |  | + | tail fiber protein | tail | NC_022968_p44 | 1.27E-161 | 500 | 0.68 |
| MACY_248 | 120930 | 121406 |  | + | tail fiber assembly protein | tail | p240703 VI_01691 | 5.71E-22 | 86 | 0.319 |
| MACY_249 | 121418 | 121732 |  | + | hypothetical protein | unknown function | KT001917_p30 | 2.09E-63 | 202 | 0.917 |
| MACY_250 | 121729 | 122202 |  | + | hypothetical protein | unknown function | NC_025830_p103 | 1.98E-100 | 312 | 0.939 |
| MACY_251 | 122216 | 122491 |  | + | membrane protein | moron, auxiliary metabolic gene and host takeover | p127674 VI_06607 | 6.46E-27 | 96 | 0.645 |
| MACY_252 | 122503 | 124263 |  | + | tail fiber protein | tail | MF979563_p34 | 1.14E-139 | 454 | 0.51 |
| MACY_253 | 124783 | 124298 |  | - | HNH endonuclease | DNA, RNA and nucleotide metabolism | p180900 VI_03586 | 6.62E-27 | 97 | 0.559 |
| MACY_254 | 126000 | 124810 |  | - | hypothetical protein | unknown function | NC_020201_p2 | 1.62E-175 | 542 | 0.672 |
| MACY_255 | 126352 | 126014 |  | - | hypothetical protein | unknown function | KT001917_p193 | 2.63E-60 | 194 | 0.793 |
| MACY_256 | 126627 | 126409 |  | - | hypothetical protein | unknown function | NC_028670_p22 | 3.48E-15 | 61 | 0.749 |
| MACY_257 | 127041 | 126637 |  | - | hypothetical protein | unknown function | NC_022968_p33 | 1.19E-14 | 64 | 0.876 |
| MACY_258 | 127345 | 127034 |  | - | hypothetical protein | unknown function | NC_022968_p32 | 9.56E-58 | 185 | 0.938 |
| MACY_259 | 127619 | 127335 |  | - | hypothetical protein | unknown function | KY652726_p12 | 8.69E-52 | 168 | 0.851 |
| MACY_260 | 127978 | 127610 |  | - | hypothetical protein | unknown function | KY652726_p11 | 5.25E-74 | 233 | 0.907 |
| MACY_261 | 128691 | 127975 |  | - | HNH endonuclease | DNA, RNA and nucleotide metabolism | NC_020843_p22 | 8.70E-48 | 162 | 0.48 |
| MACY_262 | 129361 | 128660 |  | - | PnuC-like nicotinamide mononucleotide transport | moron, auxiliary metabolic gene and host takeover | KY554772_p109 | 2.93E-68 | 222 | 0.525 |
| MACY_263 | 129612 | 129382 |  | - | hypothetical protein | unknown function | NC_019400_p247 | 3.91E-50 | 162 | 0.995 |
| MACY_264 | 129971 | 129609 |  | - | hypothetical protein | unknown function | NC_019527_p37 | 3.79E-09 | 47 | 0.338 |
| MACY_265 | 130471 | 130070 |  | - | ribonucleotide reductase NrdA-like | DNA, RNA and nucleotide metabolism | NC_011811_p4 | 1.15E-21 | 86 | 0.448 |
| MACY_266 | 130833 | 130423 |  | - | hypothetical protein | unknown function | NC_019400_p250 | 1.36E-93 | 291 | 0.972 |
| MACY_267 | 131185 | 130820 |  | - | hypothetical protein | unknown function | KY555143_p285 | 4.19E-51 | 169 | 0.603 |
| MACY_268 | 132257 | 131172 |  | - | nicotinamide-nucleotide adenylyltransferase | moron, auxiliary metabolic gene and host takeover | KT919973_p91 | 1.20E-120 | 379 | 0.613 |
| MACY_269 | 132475 | 132257 |  | - | hypothetical protein | unknown function | NC_019400_p252 | 2.51E-22 | 83 | 0.492 |
| MACY_270 | 132709 | 132497 |  | - | hypothetical protein | unknown function | KY652726_p261 | 2.79E-36 | 122 | 0.859 |
| MACY_271 | 132914 | 132720 |  | - | hypothetical protein | unknown function | KY652726_p260 | 4.39E-40 | 132 | 0.979 |
| MACY_272 | 133343 | 132987 |  | - | hypothetical protein | unknown function | NC_019401_p401 | 1.31E-48 | 160 | 0.68 |
| MACY_273 | 133783 | 133388 |  | - | hypothetical protein | unknown function | MG983743_p54 | 4.56E-48 | 160 | 0.603 |
| MACY_274 | 135864 | 133834 |  | - | hypothetical protein | unknown function | MG983743_p55 | 6.89E-297 | 908 | 0.689 |
| MACY_275 | 136187 | 135960 |  | - | hypothetical protein | unknown function | MG250486_p229 | 4.94E-64 | 202 | 1 |
| MACY_276 | 136822 | 136196 |  | - | HNH endonuclease | DNA, RNA and nucleotide metabolism | MG812495_p46 | 3.58E-25 | 95 | 0.342 |
| MACY_277 | 137248 | 136850 |  | - | DNA polymerase | DNA, RNA and nucleotide metabolism | KY514263_p49 | 2.17E-64 | 205 | 0.757 |
| MACY_278 | 137840 | 137349 |  | - | HNH endonuclease | DNA, RNA and nucleotide metabolism | MG812495_p46 | 8.26E-30 | 108 | 0.471 |
| MACY_279 | 140263 | 137963 |  | - | DNA polymerase | DNA, RNA and nucleotide metabolism | NC_019926_p15 | 1.18E-39 | 151 | 0.263 |
| MACY_280 | 140866 | 140393 |  | - | hypothetical protein | unknown function | NC_023717_p83 | 4.29E-21 | 83 | 0.329 |
| MACY_281 | 142906 | 140876 |  | - | DNA primase/helicase | DNA, RNA and nucleotide metabolism | NC_031029_p115 | 6.56E-52 | 187 | 0.277 |
| MACY_282 | 143553 | 142903 |  | - | DNA methyltransferase | other | KX495186_p170 | 2.87E-49 | 168 | 0.505 |
| MACY_283 | 143824 | 143606 |  | - | DNA ligase | DNA, RNA and nucleotide metabolism | NC_016071_p8 | 3.86E-15 | 62 | 0.454 |
| MACY_284 | 144108 | 143824 |  | - | hypothetical protein | unknown function | NC_022968_p8 | 1.07E-40 | 136 | 0.713 |
| MACY_285 | 144352 | 144101 |  | - | hypothetical protein | unknown function | NC_023717_p91 | 3.42E-32 | 114 | 0.791 |
| MACY_286 | 144638 | 144330 |  | - | DNA ligase | DNA, RNA and nucleotide metabolism | NC_016071_p6 | 4.60E-55 | 178 | 0.801 |
| MACY_287 | 144798 | 144619 |  | - | hypothetical protein | unknown function | NC_016071_p5 | 7.98E-30 | 103 | 0.825 |
| MACY_288 | 145150 | 144788 |  | - | hypothetical protein | unknown function | No_PHROG | No_PHROG | No_PHROG | No_PHROG |
| MACY_289 | 146466 | 145159 |  | - | helicase | DNA, RNA and nucleotide metabolism | KY514263_p80 | 5.67E-75 | 264 | 0.403 |
| MACY_290 | 147695 | 146466 |  | - | RIIB lysis inhibitor | lysis | MG696114_p269 | 7.60E-70 | 236 | 0.47 |
| MACY_291 | 147805 | 147695 |  | - | hypothetical protein | unknown function | No_PHROG | No_PHROG | No_PHROG | No_PHROG |

**Table S3.** Functional prediction of the *Raoultella* phage Sally, with supporting evidence

| **Gene** | **Start** | **Stop** | **Frame** | **Annotation** | **Category** | **mmseqs_top_hit** | **mmseqs_eVal** | **mmseqs_phrog** | **mmseqs_alnScore** | **mmseqs_seqIdentity** |
| --- | --- | --- | --- | --- | --- | --- | --- | --- | --- | --- |
| SALLY_0001 | 822 | 1 | - | DNA methyltransferase | other | 757 | 130 | 0.357 | 7.80E-37 | KX495186_p170 |
| SALLY_0002 | 938 | 849 | - | hypothetical protein | unknown function | No_PHROG | No_PHROG | No_PHROG | No_PHROG | No_PHROG |
| SALLY_0003 | 1302 | 931 | - | hypothetical protein | unknown function | No_PHROG | No_PHROG | No_PHROG | No_PHROG | No_PHROG |
| SALLY_0004 | 1805 | 1329 | - | hypothetical protein | unknown function | 824 | 98 | 0.403 | 5.08E-27 | NC_025471_p57 |
| SALLY_0005 | 3850 | 1802 | - | hypothetical protein | unknown function | 34481 | 89 | 0.501 | 3.24E-24 | NC_019725_p65 |
| SALLY_0006 | 3926 | 4483 | + | hypothetical protein | unknown function | 2776 | 51 | 0.456 | 1.21E-10 | p282559 VI_00606 |
| SALLY_0007 | 4468 | 4968 | + | DNA primase | DNA, RNA and nucleotide metabolism | 510 | 62 | 0.34 | 5.43E-13 | p276824 VI_04255 |
| SALLY_0008 | 5038 | 5439 | + | hypothetical protein | unknown function | No_PHROG | No_PHROG | No_PHROG | No_PHROG | No_PHROG |
| SALLY_0009 | 5449 | 6492 | + | exonuclease VIII | DNA, RNA and nucleotide metabolism | 412 | 96 | 0.268 | 1.59E-24 | p123465 VI_04287 |
| SALLY_0010 | 6570 | 7229 | + | Erf-like ssDNA annealing protein | DNA, RNA and nucleotide metabolism | 2324 | 99 | 0.482 | 7.62E-27 | KX815270_p34 |
| SALLY_0011 | 7232 | 7744 | + | single strand DNA binding protein | DNA, RNA and nucleotide metabolism | 44 | 121 | 0.485 | 1.59E-35 | p256451 VI_01358 |
| SALLY_0012 | 7754 | 7954 | + | transcriptional regulator | transcription regulation | 1373 | 38 | 0.382 | 3.73E-07 | NC_005857_p48 |
| SALLY_0013 | 7960 | 8214 | + | hypothetical protein | unknown function | 2243 | 48 | 0.558 | 1.43E-10 | KP869101_p150 |
| SALLY_0014 | 9452 | 8241 | - | tail fiber protein | tail | 9502 | 60 | 0.307 | 5.43E-12 | NC_028880_p39 |
| SALLY_0015 | 18943 | 9485 | - | tail protein | tail | 2589 | 1799 | 0.368 | 0 | p216626 VI_11255 |
| SALLY_0016 | 19545 | 18943 | - | tail protein | tail | 4946 | 116 | 0.444 | 6.06E-33 | p86380 VI_02810 |
| SALLY_0017 | 20297 | 19527 | - | minor tail protein | tail | 30 | 127 | 0.501 | 6.55E-37 | p413732 VI_07955 |
| SALLY_0018 | 20544 | 20416 | - | hypothetical protein | unknown function | No_PHROG | No_PHROG | No_PHROG | No_PHROG | No_PHROG |
| SALLY_0019 | 21893 | 20625 | - | minor tail protein | tail | 18 | 122 | 0.434 | 2.54E-34 | p22999 VI_12315 |
| SALLY_0020 | 22252 | 21890 | - | minor tail protein | tail | 14 | 82 | 0.42 | 5.75E-22 | NC_019721_p14 |
| SALLY_0021 | 25149 | 22297 | - | tail length tape measure protein | tail | 24 | 294 | 0.309 | 8.84E-85 | p178470 VI_05726 |
| SALLY_0022 | 25400 | 25146 | - | tail length tape measure protein | tail | 680 | 56 | 0.585 | 5.05E-14 | p64383 VI_11350 |
| SALLY_0023 | 25843 | 25454 | - | tail length tape measure protein | tail | 569 | 56 | 0.301 | 1.77E-12 | KY926791_p9 |
| SALLY_0024 | 26566 | 25892 | - | major tail protein | tail | 584 | 213 | 0.492 | 4.65E-65 | NC_027383_p27 |
| SALLY_0025 | 27018 | 26572 | - | tail terminator | connector | 179 | 57 | 0.296 | 8.37E-13 | NC_026610_p50 |
| SALLY_0026 | 27695 | 27015 | - | tail completion or Neck1 protein | connector | 169 | 77 | 0.428 | 6.80E-20 | NC_018454_p37 |
| SALLY_0027 | 28078 | 27695 | - | head closure Hc1 | connector | No_PHROG | No_PHROG | No_PHROG | No_PHROG | No_PHROG |
| SALLY_0028 | 28530 | 28081 | - | head-tail adaptor Ad1 | connector | 477 | 59 | 0.314 | 2.87E-13 | p198012 VI_06308 |
| SALLY_0029 | 29692 | 28586 | - | major head protein | head and packaging | 247 | 200 | 0.361 | 9.54E-59 | NC_012223_p10 |
| SALLY_0030 | 29812 | 29708 | - | hypothetical protein | unknown function | No_PHROG | No_PHROG | No_PHROG | No_PHROG | No_PHROG |
| SALLY_0031 | 30543 | 29845 | - | head scaffolding protein | head and packaging | 206 | 88 | 0.289 | 3.16E-22 | NC_016566_p9 |
| SALLY_0032 | 31694 | 30579 | - | head morphogenesis | head and packaging | 241 | 335 | 0.496 | 4.86E-105 | p334166 VI_07454 |
| SALLY_0033 | 33337 | 31694 | - | portal protein | head and packaging | 145 | 282 | 0.372 | 2.43E-85 | p275654 VI_09652 |
| SALLY_0034 | 34655 | 33297 | - | hypothetical protein | unknown function | 38319 | 147 | 0.348 | 4.19E-42 | p301012 VI_09013 |
| SALLY_0035 | 35138 | 34710 | - | hypothetical protein | unknown function | No_PHROG | No_PHROG | No_PHROG | No_PHROG | No_PHROG |
| SALLY_0036 | 36658 | 35180 | - | hypothetical protein | unknown function | No_PHROG | No_PHROG | No_PHROG | No_PHROG | No_PHROG |
| SALLY_0037 | 37214 | 36732 | - | terminase | head and packaging | 3064 | 139 | 0.523 | 1.49E-40 | NC_031113_p34 |
| SALLY_0038 | 37250 | 37351 | + | hypothetical protein | unknown function | No_PHROG | No_PHROG | No_PHROG | No_PHROG | No_PHROG |
| SALLY_0039 | 37492 | 37647 | + | hypothetical protein | unknown function | No_PHROG | No_PHROG | No_PHROG | No_PHROG | No_PHROG |
| SALLY_0040 | 37659 | 37766 | + | hypothetical protein | unknown function | No_PHROG | No_PHROG | No_PHROG | No_PHROG | No_PHROG |
| SALLY_0041 | 37809 | 39479 | + | DNA polymerase/primase | DNA, RNA and nucleotide metabolism | No_PHROG | No_PHROG | No_PHROG | No_PHROG | No_PHROG |
| SALLY_0042 | 39491 | 39706 | + | hypothetical protein | unknown function | 268 | 62 | 0.607 | 5.15E-16 | p434099 VI_06793 |
| SALLY_0043 | 39791 | 40378 | + | hypothetical protein | unknown function | No_PHROG | No_PHROG | No_PHROG | No_PHROG | No_PHROG |
| SALLY_0044 | 40418 | 40555 | + | hypothetical protein | unknown function | 24395 | 38 | 0.453 | 7.03E-08 | KX455876_p4 |
| SALLY_0045 | 40555 | 40866 | + | hypothetical protein | unknown function | 1972 | 36 | 0.375 | 2.39E-06 | MG208881_p15 |
| SALLY_0046 | 40870 | 41190 | + | hypothetical protein | unknown function | 19244 | 66 | 0.396 | 1.69E-16 | KF591601_p4 |
| SALLY_0047 | 41180 | 41449 | + | hypothetical protein | unknown function | No_PHROG | No_PHROG | No_PHROG | No_PHROG | No_PHROG |
| SALLY_0048 | 41446 | 41787 | + | hypothetical protein | unknown function | No_PHROG | No_PHROG | No_PHROG | No_PHROG | No_PHROG |
| SALLY_0049 | 41868 | 42122 | + | hypothetical protein | unknown function | 31710 | 46 | 0.345 | 3.36E-10 | KT367887_p22 |
| SALLY_0050 | 42163 | 42525 | + | hypothetical protein | unknown function | No_PHROG | No_PHROG | No_PHROG | No_PHROG | No_PHROG |
| SALLY_0051 | 42543 | 42818 | + | hypothetical protein | unknown function | 9201 | 48 | 0.524 | 4.22E-11 | NC_019538_p119 |
| SALLY_0052 | 42815 | 43099 | + | hypothetical protein | unknown function | No_PHROG | No_PHROG | No_PHROG | No_PHROG | No_PHROG |
| SALLY_0053 | 43096 | 43458 | + | hypothetical protein | unknown function | No_PHROG | No_PHROG | No_PHROG | No_PHROG | No_PHROG |
| SALLY_0054 | 43455 | 43706 | + | hypothetical protein | unknown function | 1972 | 57 | 0.376 | 8.49E-14 | MG208881_p15 |
| SALLY_0055 | 43703 | 44410 | + | hypothetical protein | unknown function | No_PHROG | No_PHROG | No_PHROG | No_PHROG | No_PHROG |
| SALLY_0056 | 44407 | 44646 | + | RNA-binding protein | DNA, RNA and nucleotide metabolism | 986 | 82 | 0.484 | 2.01E-22 | KX828710_p14 |
| SALLY_0057 | 45164 | 44673 | - | endolysin | lysis | 7 | 92 | 0.342 | 1.18E-23 | NC_001629_p14 |
| SALLY_0058 | 45667 | 45167 | - | Rz-like spanin | lysis | 312 | 59 | 0.382 | 3.26E-13 | NC_027388_p53 |
| SALLY_0059 | 45929 | 45651 | - | holin/anti-holin | lysis | 126 | 45 | 0.335 | 2.95E-09 | p223436 VI_03133 |
| SALLY_0060 | 46594 | 46070 | - | hypothetical protein | unknown function | 10558 | 187 | 0.558 | 6.05E-57 | NC_031081_p18 |
| SALLY_0061 | 46869 | 46591 | - | hypothetical protein | unknown function | No_PHROG | No_PHROG | No_PHROG | No_PHROG | No_PHROG |
| SALLY_0062 | 47022 | 46927 | - | hypothetical protein | unknown function | No_PHROG | No_PHROG | No_PHROG | No_PHROG | No_PHROG |
| SALLY_0063 | 47270 | 47019 | - | hypothetical protein | unknown function | 2817 | 63 | 0.503 | 5.53E-16 | KT825490_p38 |
| SALLY_0064 | 47988 | 47260 | - | DNA methyltransferase | other | 111 | 148 | 0.467 | 1.06E-43 | p64666 VI_08735 |
| SALLY_0065 | 48212 | 47985 | - | hypothetical protein | unknown function | No_PHROG | No_PHROG | No_PHROG | No_PHROG | No_PHROG |
| SALLY_0066 | 48478 | 48212 | - | hypothetical protein | unknown function | No_PHROG | No_PHROG | No_PHROG | No_PHROG | No_PHROG |


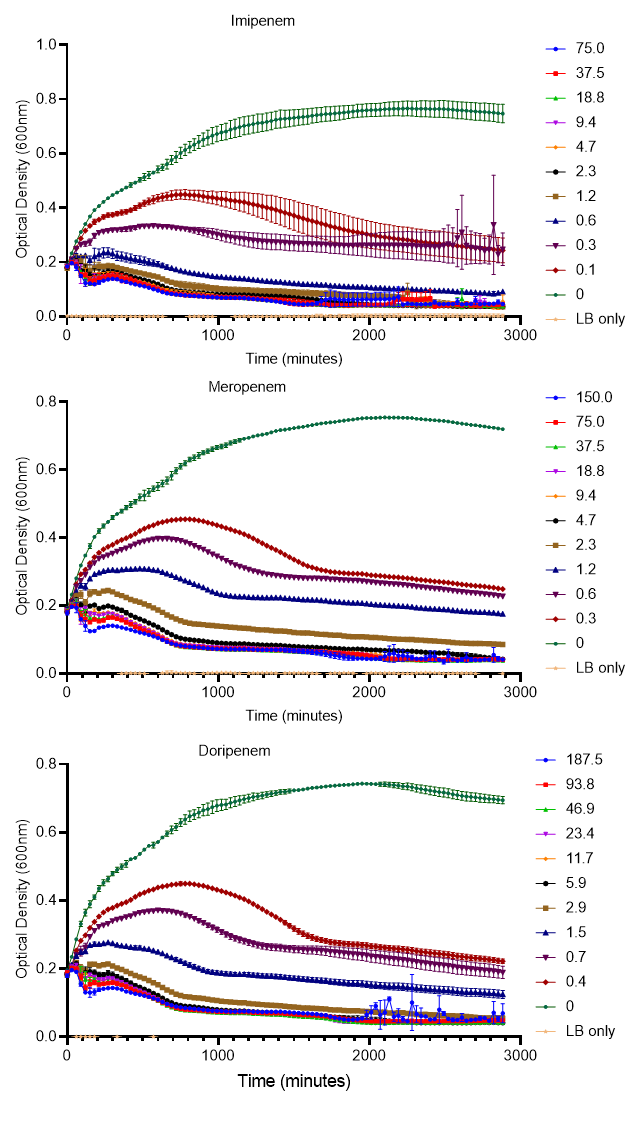


**Figure S1.** Sensitivity of *Raoultella planticola* strain RP8 to Carbapenem group of antibiotics. Bacterial growth after treatment with different concentrations of Imipenem, Doripenem, and Meropenem.


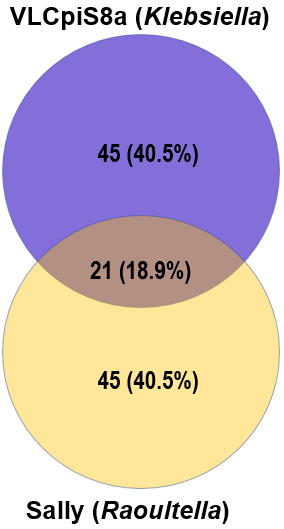


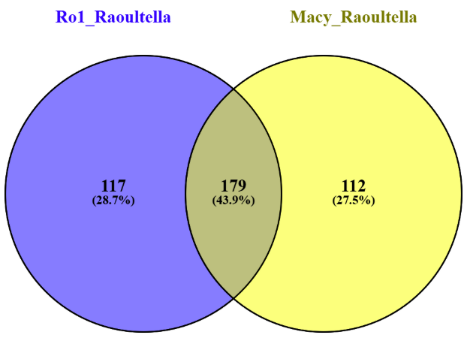


**Figure S2.** Pangenome analyses of *Raoultella* phages and closely related phages (A) Venn diagrams show the total percentages of shared and strain-specific genes between the *Raoultella* phages Macy and Ro1. (B) Venn diagram showing the number and percentages of genes shared between Sally and its closest *Klebsiella* phage VLCpiS8a.

**Table S4.** Homogenous genes between the *Raoultella* phages Macy and Ro1

| **ID** | **Source_protein_id** | **Target_protein_id** | **Identity** | **Coverage** |
| --- | --- | --- | --- | --- |
| 1 | MACY_001 | Ro1_1 | 98.24% | 81.76% |
| 2 | MACY_002 | Ro1_234 | 32.02% | 96.21% |
| 3 | MACY_003 | Ro1_3 | 52.35% | 100.00% |
| 4 | MACY_004 | Ro1_4 | 93.48% | 99.28% |
| 5 | MACY_005 | Ro1_5 | 56.45% | 103.33% |
| 6 | MACY_006 | Ro1_6 | 95.24% | 100.00% |
| 7 | MACY_007 | Ro1_7 | 98.44% | 73.85% |
| 8 | MACY_008 | Ro1_9 | 90.41% | 93.59% |
| 9 | MACY_009 | Ro1_10 | 77.14% | 100.00% |
| 10 | MACY_011 | Ro1_11 | 77.59% | 100.00% |
| 11 | MACY_012 | Ro1_12 | 84.03% | 87.27% |
| 13 | MACY_014 | Ro1_13 | 60.71% | 98.25% |
| 14 | MACY_015 | Ro1_15 | 81.36% | 100.00% |
| 15 | MACY_016 | Ro1_16 | 89.25% | 89.42% |
| 16 | MACY_017 | Ro1_17 | 77.03% | 93.67% |
| 17 | MACY_018 | Ro1_18 | 94.00% | 100.00% |
| 18 | MACY_019 | Ro1_19 | 74.51% | 94.44% |
| 20 | MACY_021 | Ro1_20 | 94.99% | 100.00% |
| 21 | MACY_022 | Ro1_21 | 82.26% | 100.81% |
| 22 | MACY_023 | Ro1_23 | 95.88% | 100.00% |
| 23 | MACY_024 | Ro1_24 | 86.77% | 99.71% |
| 24 | MACY_025 | Ro1_25 | 67.35% | 100.68% |
| 25 | MACY_026 | Ro1_26 | 91.95% | 100.00% |
| 26 | MACY_027 | Ro1_27 | 100.00% | 100.00% |
| 27 | MACY_028 | Ro1_28 | 95.59% | 100.00% |
| 28 | MACY_029 | Ro1_29 | 92.70% | 100.00% |
| 29 | MACY_030 | Ro1_30 | 96.77% | 100.00% |
| 30 | MACY_031 | Ro1_31 | 88.51% | 100.00% |
| 31 | MACY_032 | Ro1_32 | 62.56% | 98.14% |
| 32 | MACY_033 | Ro1_33 | 84.82% | 100.00% |
| 33 | MACY_034 | Ro1_34 | 97.44% | 100.00% |
| 34 | MACY_035 | Ro1_35 | 89.05% | 100.00% |
| 35 | MACY_036 | Ro1_36 | 88.68% | 100.00% |
| 36 | MACY_037 | Ro1_37 | 87.04% | 96.09% |
| 37 | MACY_038 | Ro1_39 | 90.32% | 100.00% |
| 39 | MACY_040 | Ro1_40 | 96.43% | 100.00% |
| 40 | MACY_041 | Ro1_41 | 98.47% | 100.00% |
| 43 | MACY_044 | Ro1_42 | 75.58% | 100.00% |
| 44 | MACY_045 | Ro1_43 | 95.31% | 100.00% |
| 45 | MACY_046 | Ro1_44 | 93.02% | 100.00% |
| 46 | MACY_047 | Ro1_45 | 98.61% | 100.00% |
| 47 | MACY_048 | Ro1_46 | 100.00% | 100.00% |
| 48 | MACY_049 | Ro1_47 | 98.63% | 100.00% |
| 49 | MACY_050 | Ro1_48 | 97.06% | 100.00% |
| 50 | MACY_051 | Ro1_49 | 94.44% | 100.00% |
| 51 | MACY_052 | Ro1_50 | 95.33% | 100.00% |
| 63 | MACY_065 | Ro1_56 | 89.55% | 98.53% |
| 65 | MACY_068 | Ro1_60 | 52.87% | 100.00% |
| 66 | MACY_069 | Ro1_61 | 75.41% | 100.00% |
| 67 | MACY_070 | Ro1_62 | 98.08% | 100.00% |
| 69 | MACY_072 | Ro1_63 | 95.00% | 100.00% |
| 71 | MACY_074 | Ro1_64 | 92.45% | 100.00% |
| 72 | MACY_075 | Ro1_65 | 91.07% | 100.00% |
| 73 | MACY_076 | Ro1_66 | 73.17% | 100.00% |
| 74 | MACY_077 | Ro1_67 | 65.71% | 74.47% |
| 75 | MACY_078 | Ro1_68 | 94.00% | 100.00% |
| 78 | MACY_081 | Ro1_70 | 75.00% | 80.95% |
| 79 | MACY_082 | Ro1_72 | 98.99% | 100.00% |
| 80 | MACY_083 | Ro1_73 | 98.40% | 100.00% |
| 81 | MACY_084 | Ro1_74 | 96.88% | 100.00% |
| 83 | MACY_086 | Ro1_75 | 96.61% | 100.00% |
| 84 | MACY_087 | Ro1_76 | 100.00% | 100.00% |
| 85 | MACY_088 | Ro1_77 | 94.96% | 100.00% |
| 86 | MACY_089 | Ro1_78 | 96.80% | 100.00% |
| 87 | MACY_090 | Ro1_79 | 95.12% | 100.00% |
| 89 | MACY_092 | Ro1_80 | 91.35% | 72.22% |
| 90 | MACY_093 | Ro1_8 | 35.40% | 101.26% |
| 91 | MACY_094 | Ro1_81 | 80.30% | 98.51% |
| 92 | MACY_095 | Ro1_82 | 81.76% | 98.01% |
| 93 | MACY_096 | Ro1_83 | 95.22% | 80.53% |
| 94 | MACY_097 | Ro1_85 | 64.05% | 100.00% |
| 95 | MACY_098 | Ro1_86 | 90.53% | 100.00% |
| 96 | MACY_099 | Ro1_87 | 54.72% | 100.00% |
| 98 | MACY_101 | Ro1_225 | 31.34% | 90.54% |
| 99 | MACY_102 | Ro1_88 | 96.63% | 98.89% |
| 100 | MACY_103 | Ro1_89 | 100.00% | 100.00% |
| 101 | MACY_104 | Ro1_90 | 96.93% | 100.00% |
| 102 | MACY_105 | Ro1_91 | 90.49% | 100.00% |
| 103 | MACY_106 | Ro1_92 | 96.36% | 100.00% |
| 104 | MACY_107 | Ro1_93 | 96.15% | 100.00% |
| 105 | MACY_108 | Ro1_94 | 80.09% | 98.60% |
| 107 | MACY_110 | Ro1_95 | 99.24% | 100.00% |
| 108 | MACY_111 | Ro1_96 | 100.00% | 100.00% |
| 109 | MACY_112 | Ro1_97 | 97.64% | 100.00% |
| 110 | MACY_113 | Ro1_98 | 100.00% | 100.00% |
| 111 | MACY_114 | Ro1_99 | 88.04% | 100.00% |
| 112 | MACY_115 | Ro1_100 | 88.50% | 99.12% |
| 113 | MACY_116 | Ro1_101 | 99.45% | 100.00% |
| 114 | MACY_117 | Ro1_103 | 93.54% | 74.32% |
| 115 | MACY_118 | Ro1_104 | 96.10% | 100.00% |
| 116 | MACY_119 | Ro1_105 | 100.00% | 100.00% |
| 117 | MACY_120 | Ro1_144 | 44.44% | 71.19% |
| 118 | MACY_121 | Ro1_106 | 88.82% | 103.34% |
| 119 | MACY_122 | Ro1_107 | 98.11% | 100.00% |
| 120 | MACY_123 | Ro1_108 | 98.51% | 100.00% |
| 121 | MACY_124 | Ro1_109 | 100.00% | 100.00% |
| 122 | MACY_125 | Ro1_110 | 94.55% | 100.00% |
| 123 | MACY_126 | Ro1_111 | 48.28% | 96.67% |
| 124 | MACY_127 | Ro1_116 | 32.72% | 99.69% |
| 125 | MACY_128 | Ro1_117 | 88.23% | 100.00% |
| 126 | MACY_129 | Ro1_118 | 97.52% | 99.38% |
| 127 | MACY_130 | Ro1_119 | 62.80% | 80.39% |
| 128 | MACY_131 | Ro1_120 | 97.20% | 100.00% |
| 129 | MACY_132 | Ro1_121 | 96.32% | 99.67% |
| 131 | MACY_134 | Ro1_123 | 75.00% | 97.30% |
| 132 | MACY_135 | Ro1_124 | 96.45% | 100.00% |
| 134 | MACY_137 | Ro1_125 | 96.92% | 100.00% |
| 135 | MACY_138 | Ro1_126 | 94.61% | 100.00% |
| 136 | MACY_139 | Ro1_127 | 100.00% | 100.00% |
| 137 | MACY_140 | Ro1_128 | 98.51% | 100.00% |
| 138 | MACY_141 | Ro1_129 | 94.02% | 96.69% |
| 139 | MACY_142 | Ro1_130 | 96.00% | 100.00% |
| 140 | MACY_143 | Ro1_131 | 88.05% | 100.00% |
| 141 | MACY_144 | Ro1_132 | 96.47% | 100.00% |
| 142 | MACY_145 | Ro1_133 | 98.29% | 100.00% |
| 144 | MACY_147 | Ro1_234 | 32.93% | 80.00% |
| 145 | MACY_148 | Ro1_137 | 87.53% | 97.48% |
| 147 | MACY_150 | Ro1_138 | 85.71% | 100.00% |
| 148 | MACY_151 | Ro1_139 | 47.76% | 98.53% |
| 149 | MACY_152 | Ro1_140 | 72.17% | 100.00% |
| 150 | MACY_153 | Ro1_141 | 70.55% | 101.56% |
| 151 | MACY_154 | Ro1_142 | 78.79% | 100.00% |
| 152 | MACY_155 | Ro1_143 | 74.79% | 100.00% |
| 153 | MACY_156 | Ro1_145 | 71.13% | 97.98% |
| 155 | MACY_158 | Ro1_147 | 95.29% | 89.47% |
| 156 | MACY_159 | Ro1_148 | 92.22% | 100.00% |
| 157 | MACY_160 | Ro1_149 | 91.67% | 100.00% |
| 158 | MACY_161 | Ro1_150 | 84.03% | 96.75% |
| 159 | MACY_162 | Ro1_152 | 97.00% | 100.00% |
| 160 | MACY_163 | Ro1_153 | 85.86% | 101.06% |
| 161 | MACY_164 | Ro1_154 | 90.63% | 100.00% |
| 162 | MACY_165 | Ro1_155 | 87.32% | 100.00% |
| 163 | MACY_166 | Ro1_156 | 98.78% | 100.00% |
| 164 | MACY_167 | Ro1_157 | 95.00% | 100.00% |
| 165 | MACY_168 | Ro1_158 | 100.00% | 100.00% |
| 166 | MACY_169 | Ro1_159 | 82.54% | 100.00% |
| 167 | MACY_170 | Ro1_160 | 69.56% | 100.00% |
| 168 | MACY_171 | Ro1_161 | 85.78% | 100.00% |
| 169 | MACY_172 | Ro1_162 | 97.22% | 100.00% |
| 170 | MACY_173 | Ro1_164 | 100.00% | 100.00% |
| 171 | MACY_174 | Ro1_165 | 93.64% | 100.00% |
| 172 | MACY_175 | Ro1_166 | 94.34% | 100.00% |
| 173 | MACY_176 | Ro1_169 | 98.40% | 89.92% |
| 174 | MACY_177 | Ro1_170 | 100.00% | 100.00% |
| 175 | MACY_178 | Ro1_171 | 98.97% | 100.00% |
| 176 | MACY_179 | Ro1_172 | 85.19% | 100.98% |
| 177 | MACY_180 | Ro1_173 | 95.35% | 100.39% |
| 178 | MACY_181 | Ro1_174 | 97.61% | 100.00% |
| 179 | MACY_182 | Ro1_175 | 93.09% | 100.00% |
| 180 | MACY_183 | Ro1_176 | 96.45% | 100.00% |
| 181 | MACY_184 | Ro1_177 | 98.22% | 100.00% |
| 182 | MACY_185 | Ro1_178 | 90.17% | 100.00% |
| 183 | MACY_186 | Ro1_179 | 63.36% | 100.00% |
| 184 | MACY_187 | Ro1_180 | 96.24% | 100.00% |
| 185 | MACY_188 | Ro1_181 | 98.83% | 98.84% |
| 186 | MACY_189 | Ro1_182 | 98.73% | 100.00% |
| 187 | MACY_190 | Ro1_183 | 98.63% | 100.00% |
| 188 | MACY_191 | Ro1_184 | 99.45% | 100.00% |
| 189 | MACY_192 | Ro1_185 | 93.39% | 100.00% |
| 191 | MACY_194 | Ro1_188 | 100.00% | 100.00% |
| 192 | MACY_195 | Ro1_189 | 100.00% | 86.27% |
| 193 | MACY_196 | Ro1_190 | 93.90% | 100.12% |
| 194 | MACY_197 | Ro1_191 | 93.13% | 100.00% |
| 195 | MACY_198 | Ro1_192 | 95.69% | 99.15% |
| 196 | MACY_199 | Ro1_193 | 95.12% | 99.39% |
| 197 | MACY_200 | Ro1_194 | 92.03% | 96.58% |
| 198 | MACY_201 | Ro1_195 | 96.10% | 100.00% |
| 202 | MACY_205 | Ro1_202 | 86.40% | 97.49% |
| 204 | MACY_207 | Ro1_203 | 96.79% | 100.00% |
| 205 | MACY_208 | Ro1_204 | 98.09% | 100.00% |
| 206 | MACY_209 | Ro1_205 | 74.56% | 96.85% |
| 207 | MACY_210 | Ro1_206 | 70.89% | 99.37% |
| 208 | MACY_211 | Ro1_207 | 96.19% | 100.00% |
| 209 | MACY_212 | Ro1_208 | 93.67% | 100.00% |
| 212 | MACY_215 | Ro1_71 | 31.85% | 85.44% |
| 213 | MACY_216 | Ro1_213 | 90.45% | 100.25% |
| 214 | MACY_217 | Ro1_214 | 93.81% | 100.00% |
| 217 | MACY_220 | Ro1_216 | 83.65% | 100.00% |
| 218 | MACY_221 | Ro1_217 | 80.00% | 100.00% |
| 219 | MACY_222 | Ro1_218 | 68.55% | 100.81% |
| 220 | MACY_223 | Ro1_219 | 97.36% | 98.27% |
| 221 | MACY_224 | Ro1_220 | 99.14% | 100.00% |
| 222 | MACY_225 | Ro1_221 | 74.67% | 97.40% |
| 225 | MACY_228 | Ro1_222 | 93.81% | 82.20% |
| 228 | MACY_231 | Ro1_225 | 85.54% | 89.78% |
| 229 | MACY_232 | Ro1_226 | 43.01% | 127.40% |
| 230 | MACY_233 | Ro1_227 | 65.67% | 94.37% |
| 232 | MACY_235 | Ro1_228 | 100.00% | 96.64% |
| 233 | MACY_236 | Ro1_229 | 97.73% | 100.00% |
| 234 | MACY_237 | Ro1_230 | 98.52% | 100.00% |
| 235 | MACY_238 | Ro1_231 | 97.37% | 100.00% |
| 236 | MACY_239 | Ro1_232 | 83.49% | 101.44% |
| 237 | MACY_240 | Ro1_233 | 41.18% | 89.47% |
| 239 | MACY_242 | Ro1_233 | 93.20% | 99.74% |
| 241 | MACY_244 | Ro1_235 | 95.93% | 76.67% |
| 242 | MACY_245 | Ro1_238 | 86.58% | 100.00% |
| 243 | MACY_246 | Ro1_239 | 63.01% | 100.00% |
| 244 | MACY_247 | Ro1_240 | 94.74% | 100.00% |
| 245 | MACY_248 | Ro1_241 | 98.73% | 100.00% |
| 246 | MACY_249 | Ro1_243 | 87.25% | 99.03% |
| 247 | MACY_250 | Ro1_244 | 69.23% | 86.67% |
| 249 | MACY_252 | Ro1_246 | 91.51% | 100.00% |
| 250 | MACY_253 | Ro1_247 | 98.05% | 100.00% |
| 251 | MACY_254 | Ro1_248 | 100.00% | 100.00% |
